# Stress- and mitosis-dependent phosphorylation of NIFK on Ser247

**DOI:** 10.1242/jcs.264641

**Published:** 2026-08-27

**Authors:** Sibylle Schleich, Aurelio A. Teleman

**Affiliations:** ^1^ German Cancer Research Center (DKFZ) Heidelberg, Division B140, 69120 Heidelberg, Germany; ^2^ Faculty of Medicine, Heidelberg University, 69120 Heidelberg, Germany; ^3^ Faculty of Biosciences, Heidelberg University, 69120 Heidelberg, Germany

**Keywords:** Ki-67, NIFK, Mitosis, Ribotoxic stress

## Abstract

NIFK is a nucleolar protein involved in ribosome biogenesis that interacts with Ki-67 (also known as MKI67) and is recruited to the perichromosomal layer during mitosis. NIFK expression is elevated in multiple cancers, and its expression correlates with poor prognosis. Although NIFK is highly phosphorylated, the regulation and functional significance of NIFK phosphorylation is not fully understood. Here, we identify Ser247 as a stress- and mitosis-responsive phosphorylation site on NIFK. Stress induces Ser247 phosphorylation through p38 mitogen-activated protein kinases (p38 MAPKs) and MSK1 (also known as RPS6KA5). Independently of stress, Ser247 is phosphorylated during mitosis by Aurora B (AURKB). *In vitro* assays confirm that MSK1 and Aurora B directly phosphorylate Ser247. During mitosis, Ser247 phosphorylation acts as a priming event enabling extensive multisite phosphorylation of NIFK. Mutation of Ser247 (S247A) abolishes this phosphorylation cascade but does not impair recruitment of NIFK to mitotic chromosomes. Nonetheless, cells with a NIFK S247A mutation display a delay in mitotic entry. Whether S247A mutation affects the ribosome biogenesis function of NIFK is not clear. These findings reveal that Ser247 is a hub integrating stress and mitotic cues, and establish it as a priming site for mitotic hyperphosphorylation of NIFK.

## Teaser

**Summary:** Stress- and mitosis-induced phosphorylation of NIFK at Ser247 by p38–MSK1 and Aurora B, respectively, primes mitotic hyperphosphorylation and promotes timely mitotic entry.

## INTRODUCTION

Ki-67 (also known as MKI67) is a well-established marker of cell proliferation that can be detected in all phases of the cell cycle in proliferating cells, while remaining undetectable in non-proliferating (G0) cells. Although Ki-67 expression correlates tightly with proliferative activity, making it an excellent marker of cancer cells, studies have shown that Ki-67 is not required for cell proliferation itself (Sobecki et al., 2016). Instead, Ki-67 plays a structural role during mitosis, where it directs the organization of the perichromosomal layer (Booth et al., 2014), a proteinaceous sheath that coats condensed chromosomes after nuclear envelope breakdown. This layer is composed largely of nucleolar proteins, including nucleophosmin (NPM1), nucleolin (NCL), and fibrillarin (FBL), that are displaced from the nucleolus during mitosis, as well as nucleolar RNAs such as pre-ribosomal RNA (pre-rRNA) and U3 small nucleolar RNAs (snoRNAs). This perichromosomal layer acts as a biological surfactant to maintain the separation of individual chromosomes, thereby preventing chromosome aggregation and facilitating proper chromosome segregation (Cuylen et al., 2016). One of the proteins that is nucleolar in interphase cells and associates with Ki-67 in the perichromosomal layer during mitosis is NIFK (nucleolar protein interacting with the FHA domain of Ki-67) (Takagi et al., 2001).

During interphase, NIFK localizes to the nucleolus, where it plays a role in ribosome biogenesis. NIFK promotes the maturation of 28S and 5.8S rRNAs by stimulating cleavage of the internal transcribed spacer 1 (ITS1) within pre-rRNA (Pan et al., 2015). Depletion of NIFK leads to impaired rRNA processing, nucleolar stress, p53 (TP53) activation, and cell cycle arrest in G1 (Pan et al., 2015). Structurally, NIFK consists of an N-terminal RNA-binding domain and a C-terminal Ki-67-binding domain. Functional studies have demonstrated that cells expressing a NIFK mutant lacking the Ki-67-binding domain proliferate at roughly half the speed of control cells (Pan et al., 2015). Thus, the interaction of NIFK with Ki-67 does not appear to be essential for cell viability, but it may affect the function of NIFK in ribosome biogenesis and cell cycle progression. Further work will be necessary to better understand the functional consequences of the NIFK–Ki-67 interaction.

During mitosis, NIFK undergoes extensive phosphorylation, which can be seen as a pronounced electrophoretic mobility shift by SDS-PAGE when comparing lysates from mitotic and interphase cells. This shift is abolished when phosphatase inhibitors are omitted during lysis, indicating that the modification is due to phosphorylation (Takagi et al., 2001). One key regulatory site is Thr238, which is phosphorylated by CDK1 and primes NIFK for subsequent phosphorylation at Thr234 and Ser230 by GSK3β (Byeon et al., 2005), consistent with the requirement of a phospho-residue at the +4 position for GSK3β substrate recognition. Phosphorylation at Thr234 and Thr238 enhances NIFK binding affinity for Ki-67, with Thr234 phosphorylation contributing most strongly (Byeon et al., 2005; Takagi et al., 2001). Although these findings provide clear mechanistic insights, they were derived from biochemical assays performed *in vitro* using recombinant fragments; the effect of NIFK phosphorylation on Ki-67 binding *in vivo* in cells has not been studied. It is also unclear whether the direct physical interaction between NIFK and Ki-67 is necessary for the recruitment of NIFK to the perichromosomal layer surrounding mitotic chromosomes. In the absence of Ki-67, the entire perichromosomal layer does not form (Booth et al., 2014), hence NIFK also does not localize to mitotic chromosomes. This could be due in part to direct binding between NIFK and Ki-67, and in part to binding of NIFK to other components of the perichromosomal layer.

Emerging evidence suggests that NIFK contributes to tumor progression in multiple cancer types. In lung cancer, elevated NIFK expression correlates with poor prognosis and promotes cell invasion and metastasis in functional assays (Lin et al., 2016). Similarly, NIFK is upregulated in head and neck squamous cell carcinoma, where its expression serves as a prognostic marker (Li et al., 2022). In colorectal cancer (CRC), NIFK levels are markedly increased and correlate with reduced patient survival (Tan et al., 2023; Xia et al., 2023). Knockdown of NIFK in CRC cells reduces the expression of key metabolic enzymes, including acetyl-CoA carboxylase (ACC), ATP-citrate lyase (ACLY) and fatty acid synthase (FASN), as well as the oncogenic transcription factor MYC (Xia et al., 2023). More broadly, analyses of cancer transcriptomic datasets, including GEPIA (Tang et al., 2017) and recent pan-cancer profiling studies (Li et al., 2024), indicate that NIFK is overexpressed in many tumor types and carries significant prognostic value. These findings position NIFK as both a functional effector of cellular growth and a potential biomarker in cancer.

Here we study the phosphorylation of NIFK. We find that NIFK is phosphorylated on Ser247 in two separate circumstances: upon cell stress and during mitosis. We find that two different kinases are responsible for phosphorylating Ser247 in these two conditions, with MSK1 (also known as RPS6KA5) acting during stress and Aurora B (AURKB) acting during mitosis. Interestingly, phosphorylation of Ser247 is required as a priming event during mitosis to phosphorylate NIFK on multiple additional sites, observable as a strong up-shift in mobility on an SDS-PAGE gel. Although phosphorylation of NIFK on Ser247, as well as the subsequent mitotic phosphorylations, are not required for NIFK localization to the perichromosomal layer, we find that cells carrying a NIFK S247A mutation are delayed in mitotic entry.

## RESULTS

### Generation of an antibody that detects NIFK phosphorylation on Ser247

In a phospho-proteomic experiment, we identified Ser247 of human NIFK as a site that becomes phosphorylated in response to stress (data not shown). We therefore generated a phospho-specific antibody to detect this site. We first tested antibody specificity. As expected, the antibody detects NIFK, because the signal was reduced when cells were treated with two independent siRNAs targeting NIFK (Fig. S1A). In this experiment, we treated cells with anisomycin, which induces ribotoxic stress, to promote NIFK Ser247 phosphorylation, as described in more detail below. To verify that the phospho-antibody specifically recognizes phosphorylation at Ser247, we immunoprecipitated either HA-tagged wild-type NIFK (HA–NIFK[WT]) or HA-tagged NIFK with a Ser247Ala mutation (HA–NIFK[S247A]) from cells and immunoblotted with the phospho-antibody [hereafter referred to as the p-NIFK(S247) antibody]. Consistent with specificity, the signal was diminished in the S247A mutant (lane 2, Fig. S1B). For this experiment, NIFK was immunoprecipitated from mitotic cells, because Ser247 phosphorylation is also induced during mitosis (described in more detail below). Moreover, the signal was abolished when the immunoprecipitate was treated with λ-phosphatase (compare lane 1 and lane 3, Fig. S1B), confirming phospho-specificity. In summary, the p-NIFK(S247) antibody detects phosphorylation of NIFK at Ser247.

### NIFK is phosphorylated on Ser247 by MSK1 in response to stress

**Fig. 1.**
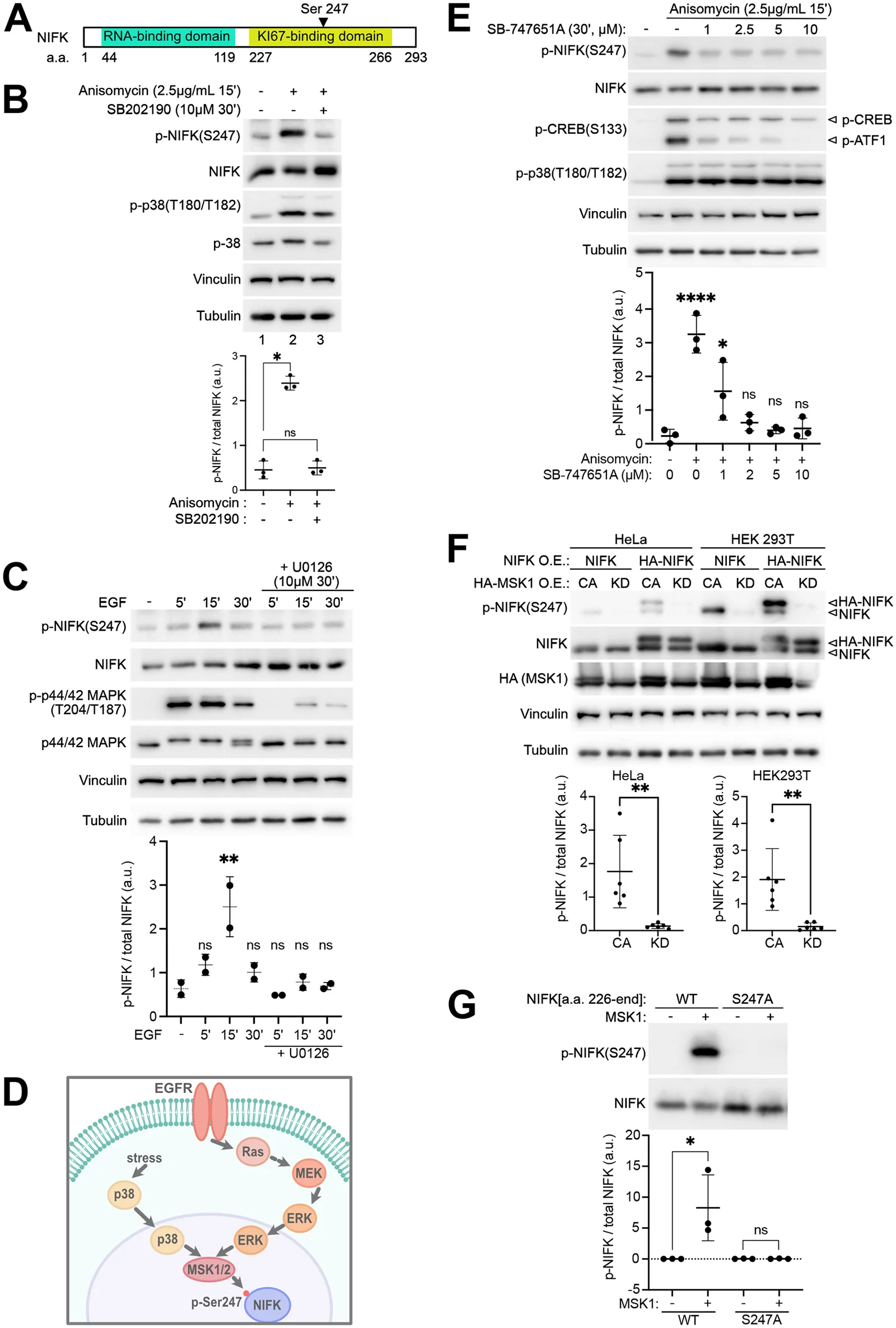
**NIFK is phosphorylated on Ser247 by MSK1 upon stress.** (A) Schematic representation of the domain structure of human NIFK and the location of Ser247 within the Ki-67-binding domain (a.a., amino acid). (B) NIFK Ser247 is phosphorylated downstream of p38 in response to ribotoxic stress caused by anisomycin. HeLa cells were either pre-treated or not with p38 inhibitor SB202190 for 30 min and then treated with anisomycin for 15 min to induce ribotoxic stress, followed by cell lysis and immunoblotting to detect the indicated proteins and phospho-proteins. Top panel: representative immunoblot. Bottom panel: quantification of NIFK Ser247 phosphorylation relative to total NIFK for *n*=3 biological replicates (a.u., arbitrary units). Mean±s.d. *P<0.05 by one-way ANOVA with Dunnett's multiple comparison testing (ns, not significant). (C) NIFK Ser247 is mildly phosphorylated in a MEK-dependent manner in response to EGF signaling. HeLa cells were either pre-treated with MEK inhibitor U0126 or not, as indicated, and then treated with 50 ng/ml EGF for the indicated times, prior to lysis and immunoblotting for the indicated proteins and phospho-proteins. Top panel: representative immunoblot. Bottom panel: quantification of NIFK Ser247 phosphorylation relative to total NIFK for *n*=2 biological replicates. Mean±s.d. **P<0.01 by one-way ANOVA with Dunnett's multiple comparison testing. (D) Schematic diagram showing that MSK1 and MSK2 (collectively referred to as MSK1/2) are activated downstream of both p38 and MEK–ERK signaling. (E) NIFK phosphorylation on Ser247 in response to anisomycin is dependent on MSK1 activation. HeLa cells were pre-treated for 30 min with the indicated concentrations of MSK1/2 inhibitor SB-747651A and then treated for 15 min with anisomycin to induce ribotoxic stress, followed by lysis and immunoblotting to detect the indicated proteins and phospho-proteins. Top panel: representative immunoblot. Bottom panel: quantification of NIFK Ser247 phosphorylation relative to total NIFK for *n*=3 biological replicates. Mean±s.d. ****P<0.001 by one-way ANOVA with Dunnett's multiple comparison testing. (F) Overexpression (O.E.) of constitutively active (CA) MSK1 but not kinase-dead (KD) MSK1 leads to phosphorylation of NIFK in cells. HeLa and HEK293T cells were transfected to express the indicated constructs, followed by lysis and immunoblotting to detect the indicated proteins and phospho-proteins. Top panel: representative immunoblot. Bottom panels: quantification of NIFK Ser247 phosphorylation relative to total NIFK. Quantifications from both HA–NIFK and non-tagged NIFK are shown together. *n*=6 biological replicates. Mean±s.d. **P<0.01 by unpaired two-tailed *t*-test. (G) MSK1 directly phosphorylates NIFK on Ser247 *in vitro*. Recombinant fragments of wild-type and S247A NIFK [a.a. 226–end] were expressed in bacteria and purified, then mixed *in vitro* with active MSK1 kinase and ATP, and then analyzed by immunoblotting with phospho-NIFK-specific antibody. Top panel: representative immunoblot. Bottom panel: quantification of NIFK Ser247 phosphorylation relative to total NIFK for *n*=3 biological replicates. Mean±s.d. *P<0.05 by one-way ANOVA with Dunnett's multiple comparison testing.

NIFK Ser247 is located within the Ki-67-binding domain of NIFK (Fig. 1A). Analysis of the PhosphoSite database (Hornbeck et al., 2015) indicated that phosphorylation at this site may be regulated by the p38 mitogen-activated protein kinase (MAPK) inhibitor SB202190, epidermal growth factor (EGF), the MEK1/2 (MAP2K1/MAP2K2) inhibitor U0126, and the Aurora A (AURKA) inhibitor MLN8054. We focused first on p38 MAPKs because the p38 kinases are activated by stress (Vind et al., 2020; Wek, 2018). Consistent with this, treatment of cells with anisomycin at a concentration that partially inhibits translation – and thereby promotes ribosome collisions to activate p38 kinases (Wu et al., 2020) – induced Ser247 phosphorylation (lanes 1 and 2, Fig. 1B). This phosphorylation was attenuated by the p38 inhibitor SB202190 (lane 3, Fig. 1B) indicating that it was mediated by p38 or a downstream kinase.

We next examined the EGFR–MAPK signaling pathway, which the PhosphoSite database also predicts to regulate Ser247 phosphorylation. Stimulation of cells with EGF led to a mild increase in NIFK Ser247 phosphorylation within 15 min, and this was blunted by the MEK1/2 inhibitor U0126 (Fig. 1C). Taken together with the p38 data (Fig. 1B), these results are intriguing because they indicate that two parallel MAPK pathways, p38 and ERK (herein referring to ERK1 and EKR2, also known as MAPK3 and MAPK1, respectively), both lead to NIFK Ser247 phosphorylation, although these two pathways tend to act in opposing ways: the p38 pathway is induced by cell stress, whereas the ERK pathway is mitogenic and induced by growth factors.

Interestingly, the mitogen- and stress-activated protein kinases MSK1 and MSK2 (also known as RPS6KA4) are activated downstream of both p38 and ERK (Fig. 1D) (Duda and Frödin, 2013). We therefore tested whether MSK1 or MSK2 might be the downstream kinases that phosphorylate NIFK on Ser247. Indeed, treatment with the MSK1 inhibitor SB-747651A markedly reduced anisomycin-induced NIFK Ser247 phosphorylation (Fig. 1E). In contrast, inhibitors of MAPK-activated protein kinase 2 (MK2, also known as MAPKAPK2; PF3644022) or of MNK1 and MNK2 (also known as MKNK1 and MKNK2, respectively; eFT508) – two other kinases that, like MSK1, are activated downstream of p38 and/or ERK kinases (Han et al., 2020; Morgan et al., 2022) – had little effect (Fig. S1C). The MSK2 inhibitor APIO-EE-07 also produced only a minor reduction in Ser247 phosphorylation (Fig. S1D) suggesting that NIFK Ser247 phosphorylation is mainly downstream of MSK1 in these cells. Consistent with this, expression of constitutively active (CA) MSK1, but not of kinase-dead (KD) MSK1, caused increased phosphorylation of NIFK on Ser247 (Fig. 1F). To test whether MSK1 can directly phosphorylate NIFK on Ser247, we performed *in vitro* kinase assays using a soluble fragment of NIFK (from amino acid 226 to the C terminus) expressed and purified from *Escherichia coli* (Fig. S1E). MSK1 efficiently phosphorylated wild-type NIFK but not S247A mutant NIFK (Fig. 1G), demonstrating that Ser247 is a direct target site of MSK1. In agreement with this, the sequence surrounding Ser247 (R-R-K-pS) perfectly matches the consensus phosphorylation motif of MSK1 (R-R-x-S or R-K-S; Duda and Frödin, 2013).

**Fig. 2.**
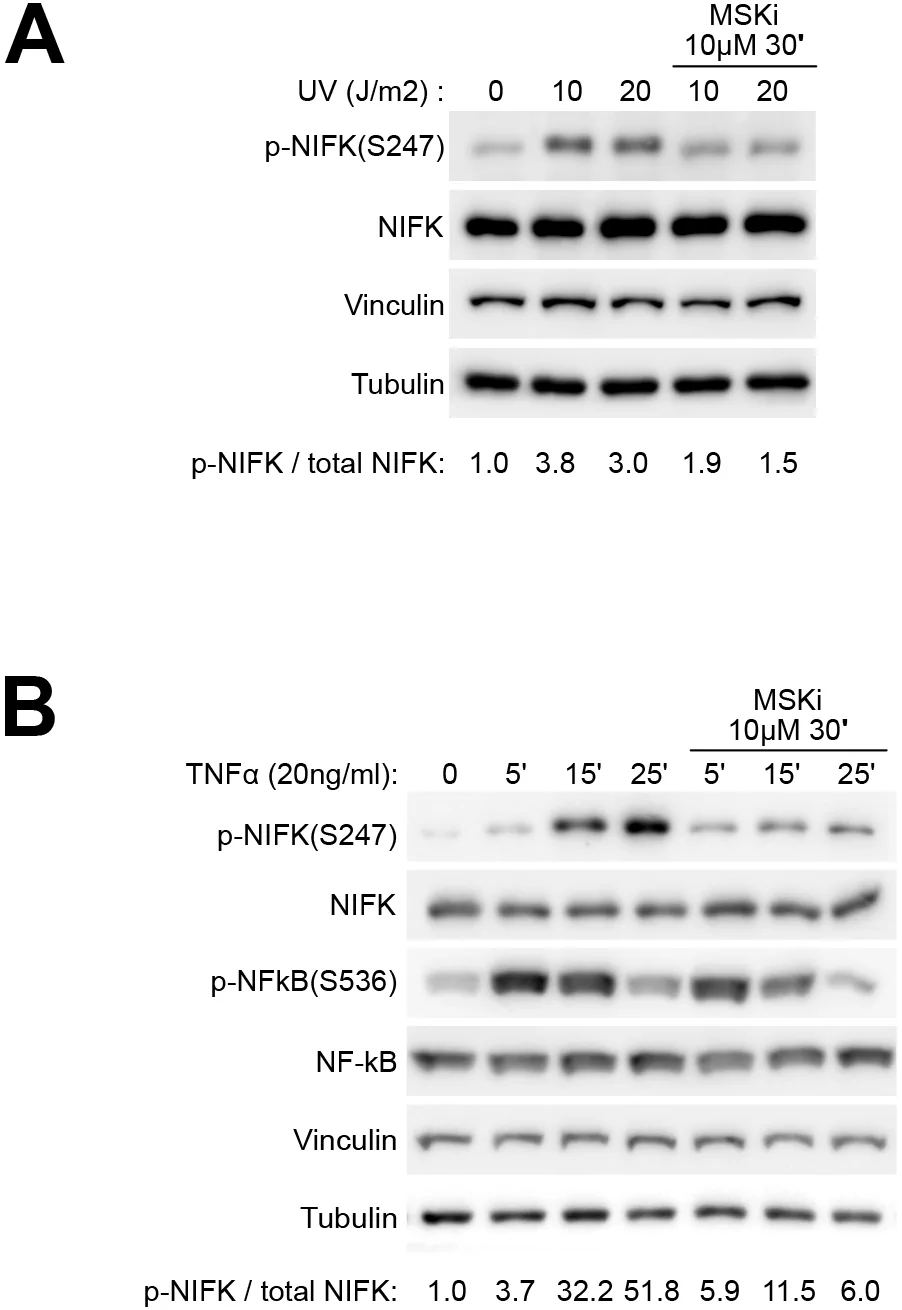
**NIFK is phosphorylated on Ser247 by MSK1 in response to UV and TNFα.** (A) UV irradiation induces NIFK Ser247 phosphorylation via MSK1. HeLa cells were pre-treated with or without MSK inhibitor (MSKi) for 30 min, as indicated, then exposed to 10 or 20 J/m^2^ UV-B before being allowed to recover for 30 min, and finally lysed and analyzed by immunoblotting to detect the indicated proteins and phospho-proteins. (B) TNFα induces NIFK Ser247 phosphorylation via MSK1. HeLa cells were treated with TNFα for the indicated timepoints, either in the presence or absence of MSK inhibitor, as indicated, before being lysed and analyzed by immunoblotting to detect the indicated proteins and phospho-proteins. Blots shown in A and B are representative of two biological replicates each. Quantification of phosphorylated NIFK Ser247 relative to total NIFK shown in A and B is for the replicate shown in the figure.

Since p38, and hence MSK1, are activated by multiple different stresses and cytokines, we tested whether additional regulatory inputs can induce NIFK phosphorylation on Ser247. Indeed, UV irradiation and treatment with TNFα (also known as TNF), both of which activate p38 (She et al., 2000; Warmerdam et al., 2013; Zarubin and Han, 2005), caused a clear increase in NIFK phosphorylation, which was blunted by MSK1 inhibitor (Fig. 2A,B).

In sum, stimuli that activate MSK1, either due to stress-dependent activation of p38 or due to growth-factor stimulation of ERK, lead to phosphorylation of NIFK on Ser247 (Fig. 1D).

### NIFK Ser247 is phosphorylated by Aurora B during mitosis

**Fig. 3.**
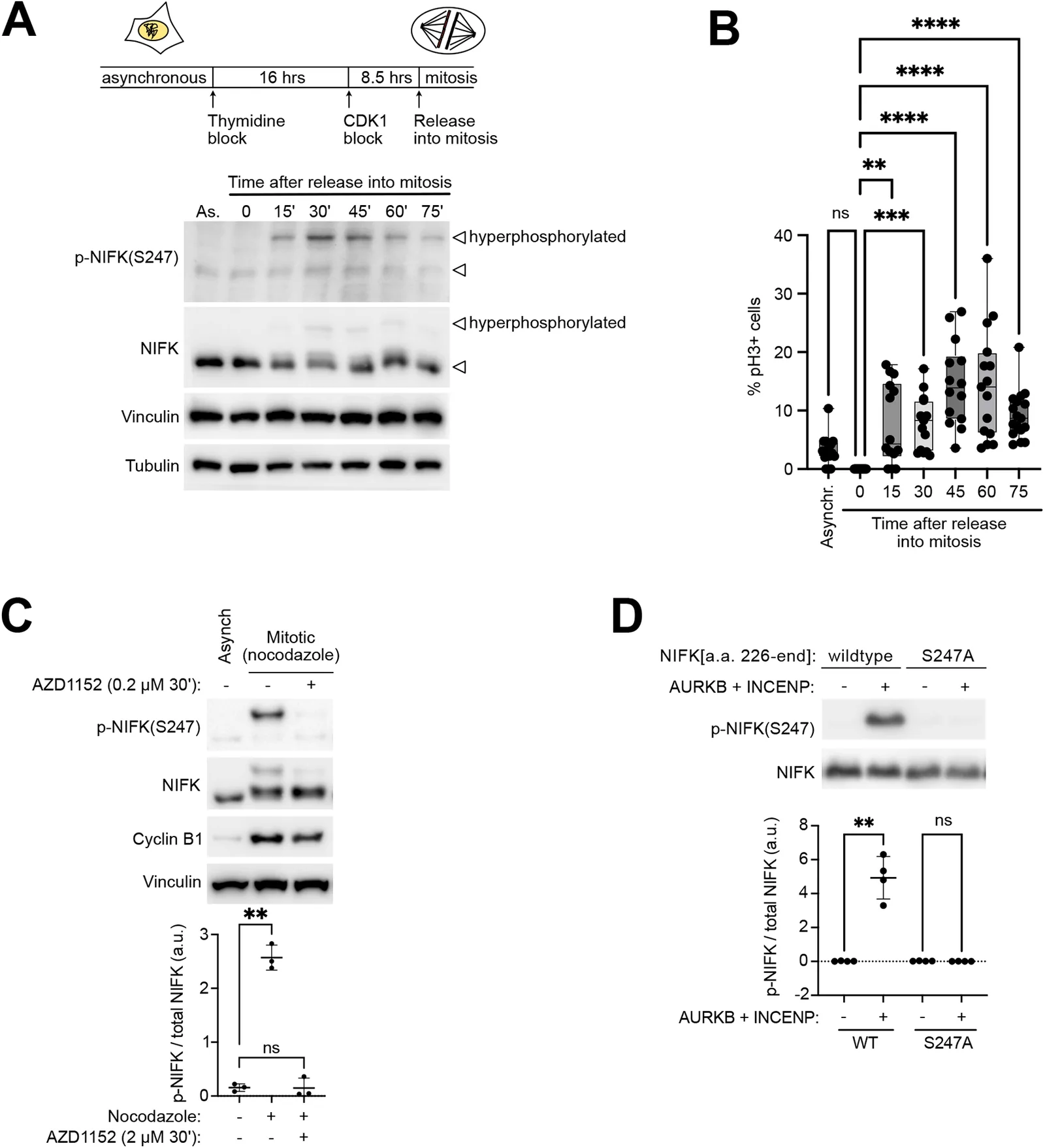
**NIFK is phosphorylated on Ser247 by Aurora B during mitosis.** (A,B) NIFK is phosphorylated on Ser247 during mitosis and displays an up-shifted electrophoretic mobility caused by phosphorylation (Fig. S1B). HeLa cells were synchronized via an overnight thymidine block, followed by release into medium containing a CDK1 inhibitor to arrest cells at the entry into mitosis, followed by release into mitosis by washout of the CDK1 inhibitor. Samples were collected at the indicated timepoints after release into mitosis. (A) Immunoblot detecting NIFK phosphorylation, total NIFK, and vinculin and tubulin controls. Arrowheads indicate p-NIFK and NIFK bands. Blots shown are representative of two biological replicates. As., asynchronous control cells. (B) Immunofluorescence analysis of phospho-histone H3-positive (pH3+) cells, expressed as percentage pH3+ per field of view, shows a peak at 45–60 min after release into mitosis. *n*=15 fields of view from two biological replicates. Asynchr., asynchronous control cells. Boxes show the 25–75th percentile, with the median indicated by a horizontal line. Whiskers indicate the minimum and maximum range. **P<0.01, ***P<0.001, ****P<0.0001 by one-way ANOVA with Kruskal–Wallis correction for multiple testing (ns, not significant). (C) Mitotic phosphorylation of NIFK on Ser247 is blunted upon inhibition of Aurora B with AZD1152. HeLa cells were synchronized in mitosis with nocodazole overnight and then treated or not with Aurora B inhibitor AZD1152 for 30 min prior to lysis and immunoblotting to detect the indicated proteins and phospho-proteins. Top panel: representative immunoblot. Asynch, asynchronous control cells. Bottom panel: quantification of NIFK Ser247 phosphorylation relative to total NIFK for *n*=3 biological replicates (a.u., arbitrary units). Mean±s.d. **P<0.01 by one-way ANOVA with Dunnett's multiple comparison testing. (D) Aurora B (AURKB) directly phosphorylates NIFK on Ser247 *in vitro*. Recombinant fragments of wild-type and S247A NIFK [a.a. 226–end] were expressed in and purified from bacteria, then mixed *in vitro* with ATP and active AURKB bound to its partner INCENP, before being analyzed by immunoblotting with phospho-NIFK-specific antibody. Top panel: representative immunoblot. Bottom panel: quantification of NIFK Ser247 phosphorylation relative to total NIFK for *n*=4 biological replicates. Mean±s.d. **P<0.01 by one-way ANOVA with Sidak's multiple comparison testing.

A previous proteome-wide phospho-proteomic study found that phosphorylation of NIFK at Ser247 is altered following treatment of cells with MLN8054 at a concentration that inhibits both Aurora A and Aurora B kinases (Hornbeck et al., 2015; Kettenbach et al., 2011). As both kinases are active during mitosis, we tested whether NIFK is phosphorylated on Ser247 in this phase of the cell cycle. To this end, we synchronized HeLa cells via an overnight thymidine block to arrest them in S phase, followed by release into thymidine-free medium containing the CDK1 inhibitor RO-3066 (10 μM) for 8.5 h, which arrests cells at the G2-M boundary. We then released this CDK1 block and collected samples every 15 min as cells entered and progressed through mitosis. This synchronization approach allows cells to progress through mitosis under minimal stress within ∼60 min. Using this system, we found that phosphorylation of NIFK on Ser247 increases midway through mitosis (Fig. 3A,B). Notably, although Ser247 phosphorylation per se does not produce a detectable mobility shift of NIFK in SDS–PAGE (Fig. 1B), phosphorylated NIFK detected by the p-NIFK(S247) antibody in mitotic lysates migrated more slowly than the unphosphorylated form (upper band, Fig. 3A). This is consistent with previous reports showing that NIFK is also phosphorylated on numerous additional sites during mitosis (Takagi et al., 2001), which – together with phosphorylation on Ser247 – cause a large band shift.

We next asked whether phosphorylation of NIFK on Ser247 during mitosis occurs via MSK1, as we observed above in the context of ribotoxic stress. To test this, we treated mitotic cells with inhibitors of MSK1 and MSK2; however, this did not reduce NIFK Ser247 phosphorylation (Fig. S2A). Thus, during mitosis, NIFK Ser247 phosphorylation occurs independently of MSK1 and MSK2, and is not a consequence of stress.

We therefore examined whether Aurora kinases contribute to NIFK Ser247 phosphorylation. Treatment of cells with the Aurora B inhibitor AZD1152 markedly reduced NIFK Ser247 phosphorylation in both cells arrested in mitosis by overnight nocodazole treatment (Fig. 3C) and in cells progressing through mitosis after synchronization with sequential thymidine and CDK1 inhibitor treatments (Fig. S2B). In contrast, an Aurora A inhibitor had no effect (Fig. S2C). Together, these data suggest that Aurora B is responsible for phosphorylating NIFK on Ser247 during mitosis. This is consistent with the fact that Aurora B activity peaks during metaphase and that Aurora B preferentially phosphorylates serine residues preceded by basic amino acids (Wheatley et al., 2004), as is the case for Ser247. To test this, we performed an *in vitro* kinase assay using active Aurora B and found that it can directly phosphorylate NIFK on Ser247 (Fig. 3D).

### Ser247 phosphorylation during mitosis primes subsequent phosphorylation events

**Fig. 4.**
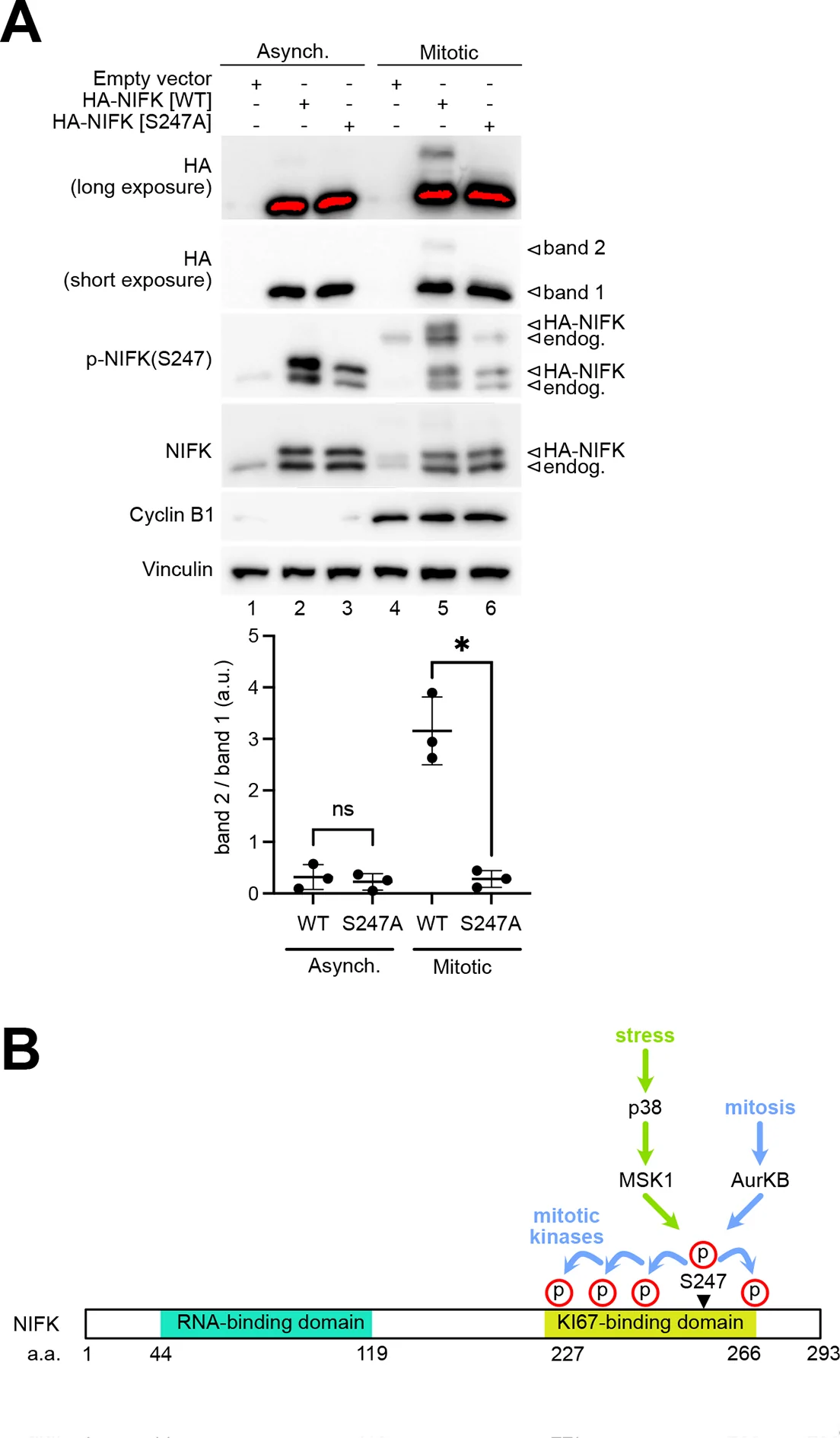
**Mitotic phosphorylation of NIFK on Ser247 is a required priming site for multiple additional phosphorylations.** (A) Mutation of NIFK Ser247 to alanine blocks the phosphorylation-dependent electrophoretic mobility up-shift of NIFK during mitosis. HeLa cells were transfected either with empty vector as a negative control, or with plasmids expressing HA-tagged wild-type NIFK (HA–NIFK[WT]) or Ser247Ala mutant NIFK (HA–NIFK[S247A]). Cells were then synchronized in mitosis via an overnight treatment with 0.1 μg/ml nocodazole before immunoblotting to detect the indicated proteins and phospho-proteins (asynch., asynchronous control cells; endog., endogenous protein). Top panel: representative immunoblot. Bottom panel: quantification of band 2 relative to band 1 for *n*=3 biological replicates (a.u., arbitrary units). Mean±s.d. *P<0.05 by one-way ANOVA with Holm–Sidak's multiple comparison testing (ns, not significant). (B) Schematic diagram summarizing the results. NIFK is phosphorylated on Ser247 in response to either ribotoxic stress via p38 and MSK1, or during mitosis via Aurora B (AurKB). During mitosis, Ser247 phosphorylation acts as a priming site for multiple additional phosphorylations on NIFK via mitotic kinases, as described previously (Takagi et al., 2001), which are not active during ribotoxic stress.

While analyzing NIFK during mitosis, we made two observations. First, overexpression of HA-tagged NIFK led to stabilization of endogenous NIFK. Since HA-tagged NIFK migrates more slowly in SDS-PAGE due to the added mass of the HA tag (lanes 1 and 2, Fig. 4A), the endogenous and exogenous forms can be distinguished. In cells expressing HA-tagged NIFK, the lower band corresponding to endogenous NIFK increased in intensity compared with that in untransfected cells (anti-NIFK, compare lane 2 and lane 1, Fig. 4A). Consistent with this, NIFK forms homodimers, as could be seen by the fact that overexpressed HA–NIFK co-immunoprecipitated endogenous NIFK (Fig. S2D). The simplest interpretation of these data is that NIFK homodimerizes, leading to its stabilization.

Second, we observed that phosphorylation at Ser247 serves as a priming event for subsequent phosphorylation events that cause the pronounced electrophoretic mobility shift of NIFK during mitosis. Specifically, we saw that endogenous NIFK shifts upward in SDS-PAGE of samples from mitotic cells (compare lane 4 and lane 1, Fig. 4A), an effect previously attributed to phosphorylation of multiple sites within the Ki-67-binding domain of NIFK by CDK1 (Takagi et al., 2001). A similar shift was seen for HA–NIFK[WT] (compare lane 5 and lane 2, Fig. 4A). In contrast, HA–NIFK[S247A] lacked this mobility shift (lane 6, Fig. 4A), indicating that phosphorylation of Ser247 is required for subsequent phosphorylation of NIFK that occurs in mitosis. The fact that these subsequent phosphorylations are CDK1 dependent also explains why NIFK does not exhibit an upward mobility shift upon Ser247 phosphorylation in interphase cells (Fig. 1).

### NIFK phosphorylation on Ser247 and subsequent phosphorylations are not needed for mitotic perichromosomal recruitment

We next asked whether Ser247-dependent hyperphosphorylation of NIFK during mitosis affects its recruitment to the perichromosomal layer, given that phosphorylation of NIFK on Thr234 and Thr238 enhances its binding affinity for Ki-67 (Byeon et al., 2005; Takagi et al., 2001). However, this was not the case. We immunostained HA–NIFK in asynchronously dividing cells and identified mitotic cells via condensation of their chromosomes. This revealed that both HA–NIFK[WT] and HA–NIFK[S247A] localize to the perichromosomal layer in metaphase cells (Fig. S3A). Two explanations are possible: (1) although phosphorylation of Ser247 is required for phosphorylation of many additional sites on NIFK during mitosis, it is not required for phosphorylation of Thr234 or Thr238; or (2) phosphorylation of Thr234 and Thr238 are indeed lost in the S247A mutant, but this does not affect recruitment of NIFK to the perichromosomal layer. To test the second possibility, we asked whether an HA-tagged NIFK with a Thr238Ala mutation (HA–NIFK[T238A]) localizes to the perichromosomal layer in mitotic cells. We tested this mutant because phosphorylation of Thr238 has been shown to be required for subsequent phosphorylation of Thr234 (Byeon et al., 2005), hence HA–NIFK[T238A] lacks phosphorylation on both Thr238 and Thr234. Indeed, mutation of either Ser247 or Thr238 abolishes the electrophoretic up-shift seen for NIFK from mitotic cells (Fig. S3B). This revealed, however, that HA–NIFK[T238A] localizes to the perichromosomal layer, just like HA–NIFK[WT] (Fig. S3C). Consistent with this, NIFK was still seen to localize to the perichromosomal layer in cells treated with Aurora B inhibitor for 20 min, which causes mitotic cells (asterisks in Fig. S3D) to display a characteristic chromosomal phenotype caused by premature entry into anaphase (Fig. S3D), despite Aurora B inhibitor strongly blunting the phosphorylation of NIFK on Ser247 and the subsequent sites (Fig. 3C). Hence it appears that phosphorylation of NIFK on Thr247, Thr238 or Thr234 is not necessary for mitotic recruitment to the perichromosomal layer, perhaps because NIFK can still bind other components of the perichromosomal layer such as rRNAs.

### Cells with NIFK[S247A] have delayed mitotic entry

**Fig. 5.**
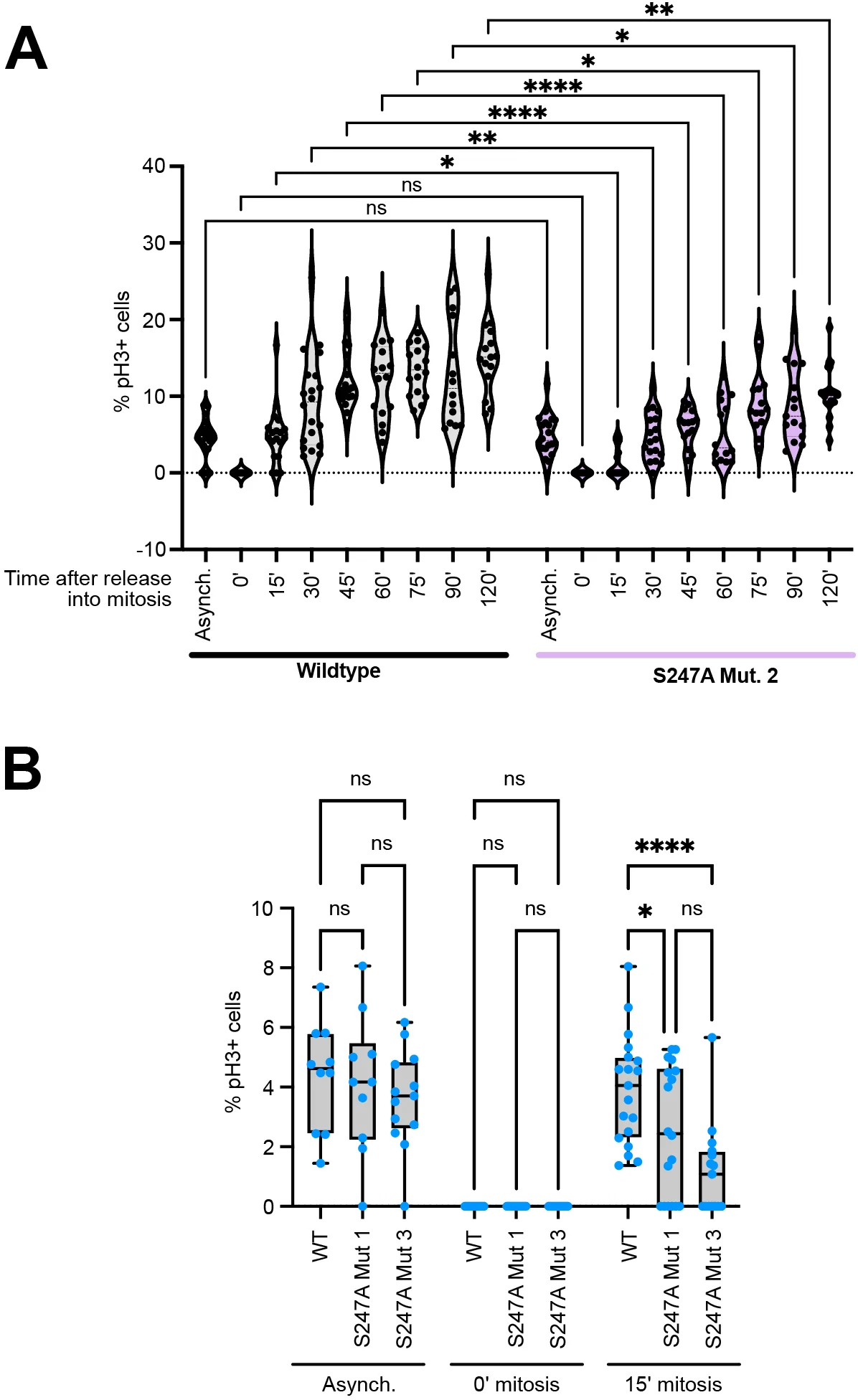
**Cells containing an S247A mutation in the endogenous NIFK locus are delayed in mitotic entry.** (A) NIFK[S247A] HEK293T cells are delayed in mitotic entry. Control (wild-type) or NIFK[S247A] (S247A Mut 2) HEK293T cells were synchronized via an overnight thymidine block then released into medium containing a CDK1 inhibitor to arrest cells at the entry into mitosis, followed by release into mitosis by washout of the CDK1 inhibitor. Samples were collected at the indicated timepoints (0–120 min) after release into mitosis then immunostained for phospho-histone H3 (pH3), and the percentage of pH3-positive cells per field of view was quantified. Asynch., asynchronous control cells. *n*=15 fields of view from one biological replicate, which is representative of three biological replicates. *P<0.05, **P<0.01, ****P<0.0001 by two-way ANOVA with Sidak's multiple comparison testing (ns, not significant). (B) The delay in mitotic entry was confirmed in two additional independent lines carrying the S247A mutation (Mut 1 and Mut 3). Control (WT) or NIFK[S247A] HEK293T cells were synchronized via an overnight thymidine block then released into medium containing a CDK1 inhibitor to arrest cells at the entry into mitosis, followed by release into mitosis by washout of the CDK1 inhibitor. Samples were collected 0 and 15 min after release into mitosis, then immunostained for phospho-histone H3 (pH3), and the percentage of pH3-positive cells per field of view was quantified. Box-and-whisker plot shows the median (middle line), 25th–75th percentile (box), and minimum to maximum range (whiskers). *n*=10 fields of view from one biological replicate, which is representative of three biological replicates. *P<0.05, ****P<0.001 by two-way ANOVA with Tukey's multiple comparison testing.

To study the functional consequences of impaired phosphorylation on Ser247, we used CRISPR-Cas9 with homology-directed repair to edit the endogenous NIFK locus to replace the Ser247 codon (TCT) with GCT, coding for alanine (Fig. S4A). We obtained three independent lines with homozygous S247A mutations (termed S247A mutant lines Mut1, Mut2 and Mut3) where NIFK cannot be phosphorylated on Ser247. Consistent with our results above, NIFK[S247A] from mitotic cells no longer exhibited an upwards mobility shift on an SDS-PAGE gel (Fig. S4B) yet it still localized to the perichromosomal layer during mitosis (Fig. S4C). Nucleolar reformation after mitosis was also unperturbed, with ∼40% of NIFK[S247A] cells showing initial NPM1 speckles in telophase cells, like control cells (Fig. S4D). Although NIFK[S247A] cells showed more chromosomal bridges in late anaphase and telophase than control cells (Fig. S4E), we did not observe micronuclei or a proliferation defect in the mutant cells, suggesting that this is not a major phenotype. We did observe, however, a delay in mitotic entry in NIFK[S247A] cells upon release from CDK1 inhibition (Fig. 5A) in all three independent mutant lines (Fig. 5A,B), suggesting that NIFK phosphorylation may contribute to the ability of cells to enter mitosis.

## DISCUSSION

We report here that NIFK is phosphorylated on Ser247 in two separate circumstances. In response to stress, it is phosphorylated by MSK1, whereas during mitosis it is phosphorylated by Aurora B (Fig. 4B). In mitosis, where CDK1 is active, phosphorylation of Ser247 leads to phosphorylation on multiple additional sites, as can be seen by a large change in NIFK migration in SDS-PAGE (Fig. 4A), which has previously been shown to be phosphorylation dependent (Takagi et al., 2001).

NIFK is known to do two things: (1) it promotes ribosome biogenesis by stimulating cleavage of the pre-rRNA ITS1 (Pan et al., 2015); and (2) it is recruited to the perichromosomal layer, which covers mitotic chromosomes, with unknown functional consequence. Does NIFK phosphorylation on Ser247 affect either of these activities? We tested whether Ser247 phosphorylation affects NIFK binding to mitotic chromosomes, but this did not seem to be the case. Indeed, phosphorylation of NIFK on Thr238 or Thr234 also did not seem to affect this (Fig. S3C). Since mutation of Ser247 to alanine blocks most of the mitotic phosphorylation of NIFK (Fig. 4A), this suggests that most of the phosphorylation sites on NIFK do not affect its recruitment to the perichromosomal layer. Nonetheless, we do observe a delay in mitotic entry in cells with a NIFK[S247A] mutation, raising the possibility that phosphorylation of NIFK plays a functional role in this context.

Does NIFK Ser247 phosphorylation affect the ribosome biogenesis function of NIFK? This would make a lot of sense, because it could act as a homeostatic feedback loop. Ribotoxic stress activates p38 kinases and MSK1, leading to NIFK phosphorylation on Ser247. If this would inhibit NIFK activity, it would dampen ribosome biogenesis, thereby alleviating the ribotoxic stress. Also, in the context of mitosis, an inhibitory function of Ser247 phosphorylation would make sense, because the nucleus and the nucleolus break down during mitosis, so ribosome biogenesis needs to be halted. Unfortunately, however, we could not obtain any data to either support or refute the hypothesis that Ser247 phosphorylation affects the ribosome biogenesis function of NIFK. One argument speaking against this hypothesis is that Pan et al. have shown that NIFK-deficient cells have a defect in cell proliferation, likely due to impaired ribosome biogenesis, and this is largely rescued by a NIFK transgene lacking the Ki-67 interaction domain, where Thr234, Thr238 and Ser247 are located (Pan et al., 2015). Thus, the Ki-67 interaction domain does not appear to be absolutely required for NIFK to promote ribosome biogenesis, although it does not rule out the possibility that this region, or Ser247 phosphorylation, could play an inhibitory role. In particular, it would seem too much of a coincidence that ribotoxic stress, of all possible inputs, regulates Ser247 phosphorylation, if this would have no functional consequence. In sum, it will be interesting to study this issue further in the future using the NIFK[S247A] genome-edited line we have generated and are happy to distribute.

## MATERIALS AND METHODS

### Immunofluorescence

HeLa or HEK293T cells were seeded on coverslips at sub-confluent concentrations and cultured overnight. The next day they were fixed in 4% paraformaldehyde (PFA) in PBS for 15 min and then washed in PBS containing 0.1% Tergitol (2×10 min). After blocking in PBS containing 0.1% Tergitol and 0.1% BSA for 30 min, the coverslips were incubated with antibody solutions in PBS containing 0.1% Tergitol and 0.1% BSA overnight at 4°C. The next day, antibody solution was washed away using PBS containing 0.1% Tergitol, and the coverslips were incubated in secondary antibody solution for 1 h. After four 10 min washes, coverslips were embedded in anti-fade solution [80% glycerol, 0.4% N-propyl-gallate (n-PG) in PBS] and imaged using a Leica SP6 confocal microscope with a Leica 63× HC PLA APO CS2 objective.

### Anisomycin-induced stress and associated drug treatments

For drug treatments, HeLa cells were treated with p38 MAPK inhibitor SB202190 (10 μM; Raybiotech/TebuBio #126331-10169-1), MK2 inhibitor PF3644022 (10 or 20 μM; Sigma PZ0188), MNK2 inhibitor eFT508 (1 or 2 μM; MedChemExpress HY-100022), MSK1 inhibitor PF-4708671 (20 μM; Medchem Express #HY-15773), MSK2 inhibitor APIO-EE-07 (5 μM; Axon Medchem Axon 3328) or MSK1/2 inhibitor SB-747651A dihydrochloride (1, 2.5, 5 or 10 μM; MedChemExpress HY-110313) for 30 min, followed by addition of 2.5 μg/ml anisomycin (Cayman/Biomol #11308) for 15 min. For inhibition of the ERK pathway, MEK1/2 inhibitor U0126 (Sigma #662005) was added at 10 μM for 30 min, followed by addition of 50 ng/ml EGF (Cell Signaling Technology #72528) for 5, 15 or 30 min (Fig. 1C), before cells were harvested by scraping in RIPA buffer (Pierce, #89900) and prepared for western blot analysis.

### Cell cycle synchronization and associated drug treatments

HeLa cells were enriched for mitotic cells either by addition of nocodazole (0.1 μg/ml; Sigma #SML1665) for 16 h or by overnight treatment with thymidine (1 mM; Sigma #T9250) followed by washing and replacement with CDK1 inhibitor RO-3066 (10 μM; Sigma # SML0569) for 8.5 h. After this, cells were released into mitosis by removing the inhibitor and briefly washing the cells. Samples were collected at the time of release (time 0) and then every 15 min. In case of inhibition of Aurora A (MLN8045, 1 μM; Raybiotech/TebuBio #126331-10894-1) or Aurora B (AZD1152, 0.2 μM; Sigma SML0268), drugs were added immediately after washout of CDK1 inhibitor and incubated for a further 30 min. At the time of harvesting, cells were briefly washed and collected by scraping in RIPA buffer, containing 1× protease inhibitors (Roche), 1× PhosStop (Roche), 2 mM Na orthovanadate, 35 mM β-glycerophosphate and 10 mM NaF for phosphatase inhibition. After 10 min incubation with benzonase at room temperature, lysates were centrifuged and mixed with Laemmli buffer for PAGE and immunodetection.

### Analysis of mitotic progression

To analyze mitotic progression, wild-type and mutant S247A HEK293T cells were synchronized by thymidine block and CDK1 inhibition as described above. After release of the G2/M block, cells were fixed with PFA at the indicated timepoints and stained for mitotic cells using anti phospho-histone H3 antibody (p-Histone 3-S10). At least ten images were taken per condition. The cell number was determined using Fiji/ImageJ (https://imagej.net/software/fiji/), and the ratio of phospho-histone H3-positive cells calculated.

### Analysis of lagging strands

To determine whether wild-type or mutant HEK293T cells show mitotic defects such as lagging chromosomes or chromosomal bridges, cells were seeded onto coverslips and, after overnight incubation, fixed with PFA, followed by DNA staining with Hoechst 33342 for 15 min. The samples were examined and scored by confocal imaging (Leica SP6, with Leica 63× HC PLA APO CS2 objective) for the presence or absence of lagging chromosomes or chromosomal bridges during late anaphase and telophase.

### Cell culture

HeLa and HEK293T cells (from DSMZ) were tested negative for mycoplasma every couple of months and were authenticated using Multiplex Cell Authentication by Multiplexion prior to freezing aliquots that were used by the lab. Cells were cultured in DMEM high glucose (Thermo Fisher Scientific) with 10% FBS (Sigma) and 1% penicillin-streptomycin (Thermo Fisher Scientific).

For transient transfections, HeLa or HEK293T cells were transiently transfected with polyethylenimine (PEI) at a PEI:DNA ratio of 3:1 and incubated for 24 h, after which they were either harvested or treated overnight with nocodazole for cell cycle synchronization.

For siRNA treatment, HeLa cells were reverse transfected with 10 pmol siRNA duplexes (Dharmacon) (Table S1) and Lipofectamine RNAiMax (Life technologies), according to the manufacturers protocol. siRNA against Renilla luciferase (RLuc) was used as a non-targeting negative control. After 3 days cells were additionally treated with anisomycin for 30 min, before harvesting in RIPA buffer.

### Radiation-induced cell stress

HeLa cells were seeded into dishes at sub-confluent density. After overnight incubation, cells were left untreated as a negative control or treated with 10 μM MSK1 inhibitor SB-747651A for 30 min. Afterwards, the medium was removed but kept. Cells were immediately exposed to UV-B light (302 nm, UVP Crosslinker, Analytik Jena) at 10 or 20 J/m^2^. The medium with or without MSK1 inhibitor was added back for further 30 min of incubation, before cells were harvested in RIPA buffer.

### TNFα-dependent phosphorylation of NIFK on Ser247

For the treatment with TNFα, Hela cells were seeded in 6-well plates at sub-confluent density and incubated overnight. The next day, cells were pre-treated for 30 min with MSK1 inhibitor SB-747651A or left untreated. Afterwards 20 ng/ml TNFα (Sigma, GF314) was added for 5, 10 or 25 min, both in control or inhibited condition, before being prepared for harvesting and western blot analysis.

### Nucleoli reformation

To quantify nucleoli reformation, wild-type or mutant S247A (Mut1) HEK293T cells were seeded onto coverslips at sub-confluent density. The next day, cells were fixed with 4% PFA and prepared for immunofluorescence. After overnight staining with anti-NPM1 antibody, cells were washed and incubated simultaneously with secondary anti-mouse IgG–FITC and Alexa Fluor 568–phalloidin, followed by 15 min incubation with Hoechst 33342. Then, the coverslips were mounted on slides and inspected under the confocal microscope. To detect cells in late telophase/cytokinesis the actomyosin ring was located and the corresponding cells were inspected for NPM1-positive speckles and quantified.

### Immunoprecipitation

HEK293T cells were transfected for 24 h with HA constructs and subsequently synchronized by overnight treatment with nocodazole or left asynchronous. The next day, cells were lysed in SDS-free RIPA buffer [25 mM Tris-HCl pH 7.5, 150 mM NaCl, 1% Tergitol 15-S-9, 1% Na desoxycholate, and protease and phosphatase inhibitors (protease inhibitor Roche, cOmplete Ultra Mini, EDTA free #05892791001, and Roche PhosStop #0406837001)], briefly treated with benzonase and centrifuged. Magnetic anti-HA beads (Pierce #88837) were added to the supernatant and rolled for 1 h at 4°C. The beads were collected using a magnetic rack and extensively washed in lysis buffer before elution into Laemmli buffer and immunoblot analysis.

### Lambda phosphatase treatment

HeLa cells were plated at a sub-confluent density. After 6 h, they were transfected with pCDNA-HA-NIFK[WT] or pCDNA-HA-NIFK[S247A] plasmids and incubated for 24 h. Next, nocodazole (0.1 μg/ml) was added for 16 h, and then cells were lysed in SDS-free RIPA buffer with benzonase and protease inhibitor, with or without phosphatase inhibitors. After brief centrifugation, magnetic anti-HA beads were added to the samples and rolled for 1 h at 4°C. After immunoprecipitation and washing in lysis buffer, as described above, the buffer was changed to PMP buffer [20 mM Tris-HCl pH 7.5, 100 mM NaCl, 0.01% Brij-35, 2 mM dithiothreitol (DTT), 1 mM MnCl_2_, 400 U λ-phosphatase (New England Biolabs)], and incubated for 30 min at 30°C. The reaction was stopped with Laemmli buffer.

### Immunoprecipitation with hypotonic lysis

HeLa cells were transfected with either empty vector (EV) or HA-NIFK[WT]. After 24 h, transfected cells were harvested by scraping in culture medium and collected by centrifugation. Cells were then lysed using a hypotonic buffer as follows (Broeck and Klinge, 2023): the pelleted cells were Dounce homogenized with seven strokes in lysis buffer A (25 mM HEPES pH 7.5, 65 mM NaCl, 65 mM KCl, 3 mM MgCl_2_, 1 mM EDTA, 10% glycerol, 0.05% Tergitol, 1 mM DTT, protease inhibitors). Pellets and supernatants were separated in a table-top centrifuge at 5000 rpm (2655 ***g***) for 5 min at 4°C. The supernatant was discarded, and the pellets were washed twice with lysis buffer A. After the second wash, nuclei were disrupted by adding hypotonic lysis buffer to the pellets (25 mM HEPES pH 7.5, 10 mM KCl, 10 mM MgCl_2_, 1 mM CaCl_2_, 1 mM EDTA, 100 mM arginine, 5 mM ATP, 1 mM spermidine, 5% glycerol, 0.1% Tergitol, 1 mM DTT, 2 U/μl RNase-free DNaseI, protease inhibitors). The suspensions were turned on a wheel for 30 min at 4°C, followed by removal of the insoluble fractions by spinning at 25,000 ***g*** for 30 min at 4°C. After adding magnetic anti-HA beads, the supernatants were turned on a wheel for 2 h at 4°C. Bound proteins were washed twice with wash buffer (25 mM HEPES pH 7.5, 75 mM NaCl, 75 mM KCl, 5 mM MgCl_2_, 0.5 mM EDTA, 100 mM arginine, 2.5% glycerol, 0.1% Tergitol, 1 mM DTT, protease inhibitors) with the help of a magnetic rack and eluted with 1× Laemmli buffer.

### Antibodies

The anti-p-NIFK(S247) antibody was generated by Davids Biotechnologie (Regensburg Germany) with animal experimentation approval. Rabbits were immunized with phosphopeptide LERRKpSQVAELNDDDKD and the final bleed was affinity purified.

Additional antibodies used in this study were: anti-total NIFK (Proteintech #12615-1-AP), anti-p-p38 MAPK T180/Y182 (Cell Signaling Technology #9211), anti-total p38 MAPK (Cell Signaling Technology #9212), anti-MAPK p-ERK1/2 p44/p42 T204/T187 (Cell Signaling Technology #5726), anti-total ERK1/2 (Cell Signaling Technology #9107), anti-HA tag (Cell Signaling Technology #3724 and #2367), anti-cyclin B1 (Cell Signaling Technology #4138), anti-p-CREB (Cell Signaling Technology #9196), anti-p-histone H3 S10 (Cell Signaling Technology #9701), anti-total histone H3 (Cell Signaling Technology #14269), anti-tubulin (Sigma T9026), anti-vinculin (Cell Signaling Technology #13901), anti-NPM1 (Life Technologies #32-5200), anti-NF-κB (Cell Signaling Technology #8242), anti-p-NF-κB S536 (Cell Signaling Technology #3033), donkey anti-rabbit IgG–FITC (Dianonva/Jackson Immuno 711-095-152), anti-mouse IgG–FITC (Dianonva/Jackson Immuno 715-095-151), and Alexa Fluor 568–phalloidin (Molecular Probes). Primary antibodies for western blotting were used at 1:1000 dilution except for anti-p-NIFK(S247), which was used at 1:500 dilution. Secondary antibodies for western blotting were used at 1:5000 dilution. For immunostaining, antibodies were used at 1:200 dilution.

### Purification of recombinant NIFK protein

Plasmids pETGB_1-NIFK[a.a.226-end][WT] and pETGB_1-NIFK[a.a.226-end][S247A] were electroporated into Rosetta pLys S bacteria (Merck, #70956-3). After overnight incubation, 200 ml of LB medium was inoculated with the pre-culture and grown to mid-log stage. The cultures were cooled to 22°C and induced with 0.5 mM IPTG. After overnight induction, cells were harvested and lysed in 50 mM HEPES 7.5, 150 mM NaCl, 20 mM imidazole, 1 mM MgCl_2_, 0.5%Tergitol, 1 mM DTT, 1× Fast Break (Promega) and EDTA-free protease inhibitors (Roche). After thorough lysis, bacteria were treated with DNAse (Sigma) and incubated at room temperature for 15 min. Afterwards, lysates were centrifuged (20,000 ***g*** for 20 min), and the supernatants were mixed with washed Ni-NTA beads (GE Healthcare) and incubated for 1 h at 4°C with rolling. Beads were then collected and washed extensively with lysis buffer without Fast Break. Bound proteins were eluted in three rounds with 300 mM imidazole, and eluates were combined. Lysis buffer without Fast Break was added to the eluates to dilute imidazole, and TEV protease (Merck, #T4455) was added to digest eluted proteins overnight at 4°C. The next day, digested His–GB1 tag was removed by adding washed Ni-NTA beads. The eluates were concentrated, aliquoted and store at −80°C until further usage.

### Plasmids

The coding sequence of NIFK was amplified using proofreading Q5 polymerase and primers NIFK-topo-fwd and NIFK-topo-rev (sequences in Table S1) on HeLa cDNA. The PCR product was A-tailed and cloned using the TOPO TA cloning kit (Life technologies). Afterwards the coding sequence was subcloned into pCDNA-HA (European Plasmid Repository, EPR#1043) via EcoRI/NotI restriction sites. The S247A mutation was introduced via overlap PCR. All vectors were verified by sanger sequencing. Plasmids can be obtained from the European Plasmid Repository (EPR#1044, EPR#1045).

For purification of NIFK protein fragments (NIFK[a.a. 226-end][WT] and NIFK[a.a. 226-end][S247A]), the plasmids described above containing the wild-type or S247A mutant NIFK were used as template, and the coding sequences were amplified with the primers NIFK aa226 fwd and NIFK Not rev (sequences in Table S1) and cloned via NcoI and NotI restriction sites into pETGB_1 (kind gift from the lab of Irmgard Sinning, BZH, Heidelberg, Germany).

Plasmids expressing constitutively active or kinase dead MSK1 were kindly provided by Peter Cheung (Lau and Cheung, 2011) and were originally made by Morten Frödin (Frödin et al., 2000).

### *In vitro* kinase assays

*In vitro* kinase reactions were performed in 8 mM MOPS (pH 7), 0.2 mM EDTA and 2 mM Na_2_ATP at 30°C for 30 min. 1 μg of purified proteins NIFK[a.a.226-end][WT] or NIFK[a.a.226-end][S247A], described above, were used per reaction. 100 ng of MAPK2-activated human MSK1 (Merk/Millipore) or full-length human Aurora B in complex with C-terminal INCENP (Merk/Millipore) was diluted in 20 mM MOPS-NaOH pH7.0, 1 mM EDTA, 5% glycerol, 0.01% Brij-35, 1 mg/ml BSA and 1 mM DTT and added to each reaction. As a positive control, 0.5 μg histone H3 was used. The reaction was stopped by the addition of 1× Laemmli buffer and subsequently heated to 95°C before western blot analysis.

### Generation of NIFK[S247A] mutant HEK293T cells

To establish HEK293T cell lines containing the S247A point mutation in NIFK, we used genome editing by CRISPR-Cas9 and homology-directed repair (HDR), using the sgRNA and HDR template specified in Table S1. To electroporate the RNP complex [Cas9, Alt-R S.p. HiFi Cas9 Nuclease V3-IDT and sgRNA, together with HDR repair template and HDR enhancer vs1 (IDT)] into the cells we used the Amaxa SF cell line 4D Nucleofector kit (Lonza). After nucleofection, cells were seeded into plates, and after 2 days of recovery, cells from the pool were genotyped by genomic PCR (Table S1). To obtain monoclonal mutant lines, single-cell selection was performed, and the lines again genotyped by PCR to confirm the mutation.

## Supplementary Material



10.1242/joces.264641_sup1 Supplementary information
