# Supplementary material for: Stress- and mitosis-dependent phosphorylation of NIFK on Ser247
Source: J Cell Sci. 2026 Aug 27;139(16):jcs264641. doi: 10.1242/jcs.264641 (PMC13571814; doi:10.1242/jcs.264641)
Supplement: Supplementary file 1 [file joces-139-264641-s1.pdf]

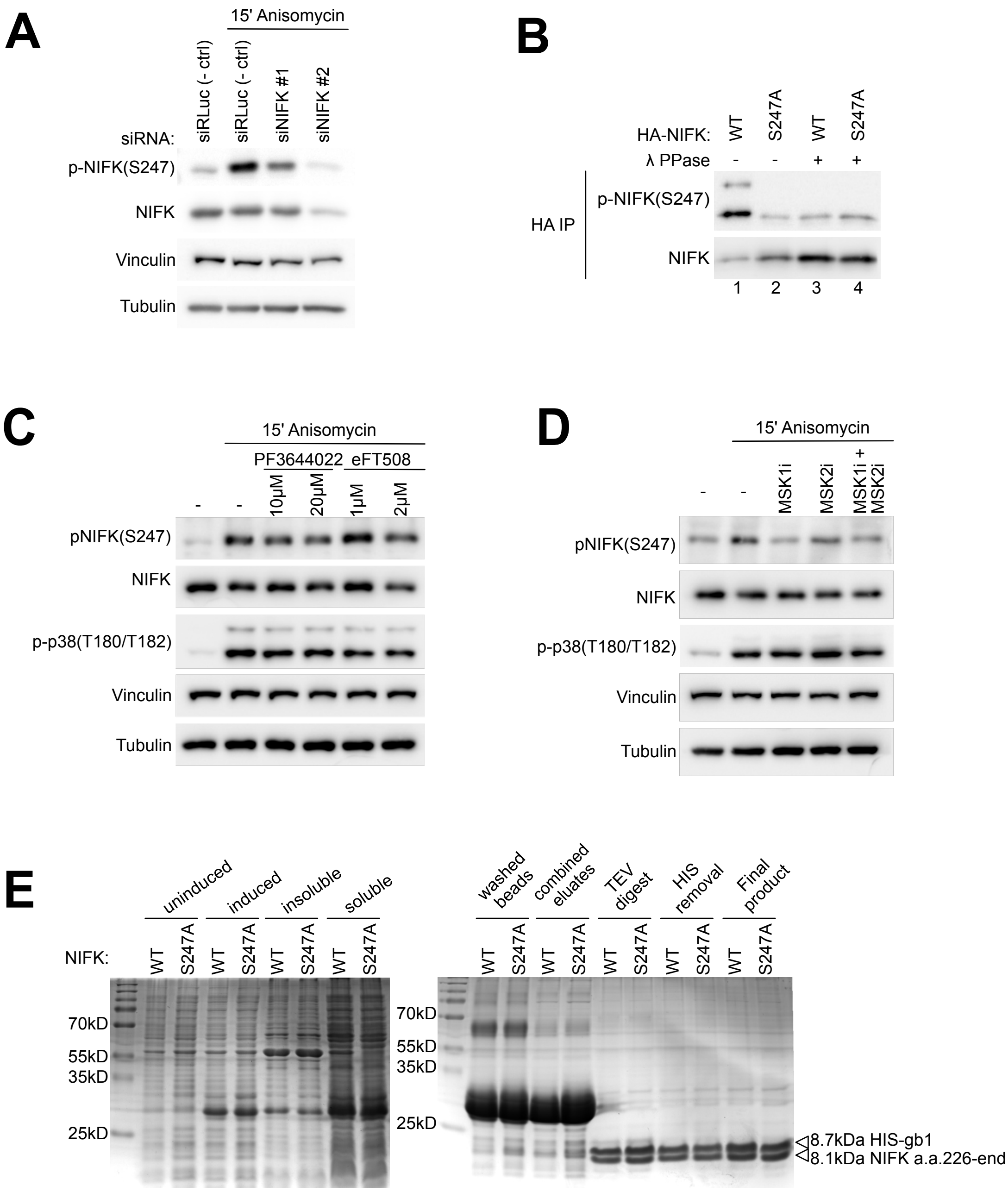

Journal of Cell Science • Supplementary information

Fig. S1. Support to main Figure 1

(A) The phospho-NIFK(S247) antibody specifically detects NIFK, as its signal is reduced upon NIFK knockdown using two independent siRNAs. HeLa cells were transfected with siRNAs targeting renilla luciferase (RLuc) as a non-targeting negative control, or with two independent siRNAs targeting NIFK. 15 minutes prior to cell harvesting, the cells were treated with anisomycin to induce NIFK phosphorylation on Ser247. Representative of 2 biological replicates.

(B) The phospho-NIFK(S247) antibody detects phosphorylation on Ser247 because its signal decreases upon mutation of Ser247 to alanine (S247A) or upon treatment with lambda phosphatase. HeLa cells were transfected to express either HA-NIFK[WT] or HA-NIFK[S247A], then synchronized in mitosis with an overnight nocodazole incubation. HA-tagged NIFK was immunoprecipitated with HA-beads. Following washing, the immunoprecipitates were incubated with or without λ-phosphatase to remove phosphorylations prior to immunoblot analysis. 1 biological replicate.

(C) Phosphorylation of NIFK on Ser247 in response to anisomycin-induced ribotoxic stress is not dependent on MK2 or MNK2 activation. HeLa cells were pre-treated with the MK2 inhibitor PF3644022 or the MNK2 inhibitor eFT508 for 30 minutes followed by induction of ribotoxic stress with anisomycin for an additional 15 minutes prior to lysis and immunoblotting. Representative of 4 biological replicates.

(D) Phosphorylation of NIFK on Ser247 in response to anisomycin-induced ribotoxic stress is reduced by the MSK1 inhibitor (MSK1i) PF-4708671 but not by the MSK2 inhibitor (MSK2i) APIO-EE-07. MCF7 cells were pre-treated with

Journal of Cell Science • Supplementary information

the indicated inhibitors for 30 minutes followed by induction of ribotoxic stress with anisomycin for an additional 15 minutes prior to lysis and immunoblotting. Representative of 2 biological replicates.

(E) Coomassie stained polyacrylamide gels showing induction and purification of NIFK[a.a.226-end][WT] or NIFK[a.a.226-end][S247A] from E. coli. After HIS-tag purification of HIS-gb1-NIFK constructions, the HIS-gb1 tag was cleaved off with TEV protease.

Journal of Cell Science • Supplementary information

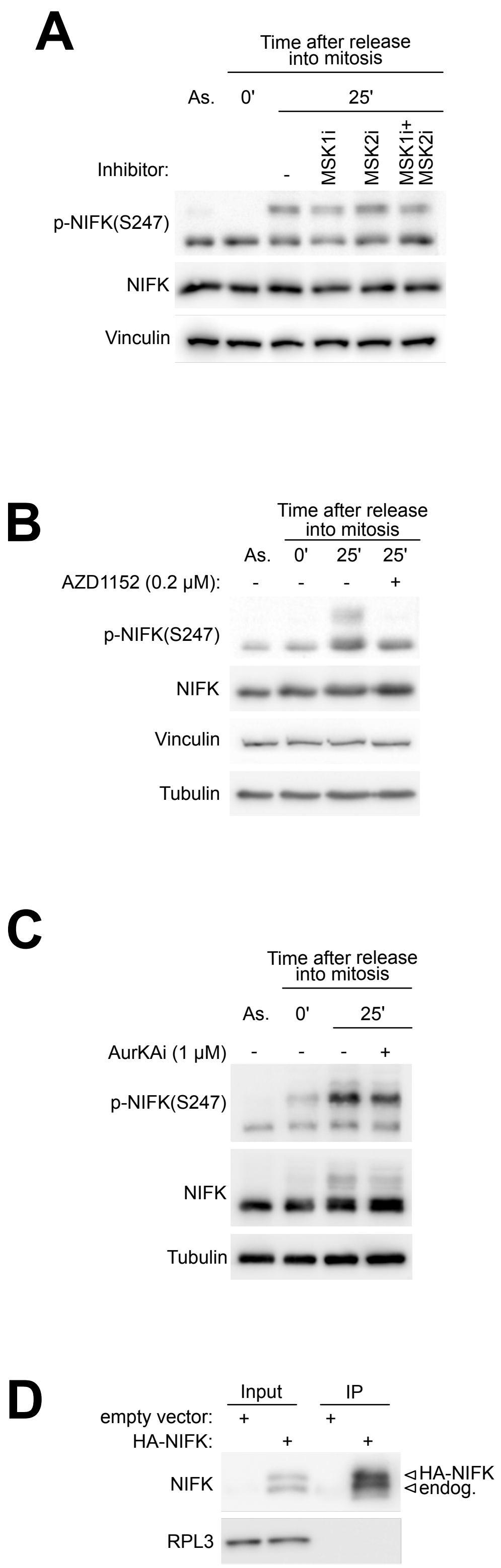

Journal of Cell Science • Supplementary information

Fig. S2. Support to Main Figure 2.

(A) Phosphorylation of NIFK on Ser247 during mitosis is not dependent on MSK1 or MSK2. HeLa cells were synchronized in mitosis via an overnight thymidine block, followed by release into medium containing Cdk1 inhibitor for 8.5 hours, followed by release into mitosis for 25 minutes. Upon release into mitosis, cells were either left untreated or treated with MSK1 inhibitor (MSK1i) PF-4708671 or MSK2 inhibitor (MSK2i) APIO-EE-07. "As." = asynchronous. Representative of 2 biological replicates.

(B) Mitotic phosphorylation of NIFK on Ser247 is blunted upon inhibition of Aurora Kinase B with AZD1152. HeLa cells were synchronized in mitosis via an overnight thymidine block, followed by release into medium containing Cdk1 inhibitor for 8.5 hours, followed by release into mitosis for 25 minutes. Upon release into mitosis, cells were either left untreated or treated with Aurora Kinase B inhibitor AZD1152. "As." = asynchronous. 1 biological replicate.

(C) Phosphorylation of NIFK on Ser247 during mitosis is not dependent on Aurora Kinase A. HeLa cells were synchronized in mitosis via an overnight thymidine block, followed by release into medium containing Cdk1 inhibitor for 8.5 hours, followed by release into mitosis for 25 minutes. 5 minutes prior to release into mitosis, cells were either left untreated or treated with Aurora Kinase A inhibitor (AurKAi) MLN8045. "As." = asynchronous. Representative of 2 biological replicates.

(D) NIFK homodimerizes. Endogenous NIFK co-immunoprecipitates with HA-NIFK[WT] in HeLa cells. HA-NIFK can be distinguished from endogenous NIFK because of its reduced mobility on an SDS-PAGE gel. Note that expression of HA-NIFK also stabilizes endogenous NIFK, leading to increased levels of both the upper and lower NIFK bands. Representative of 2 biological replicates.

Journal of Cell Science • Supplementary information

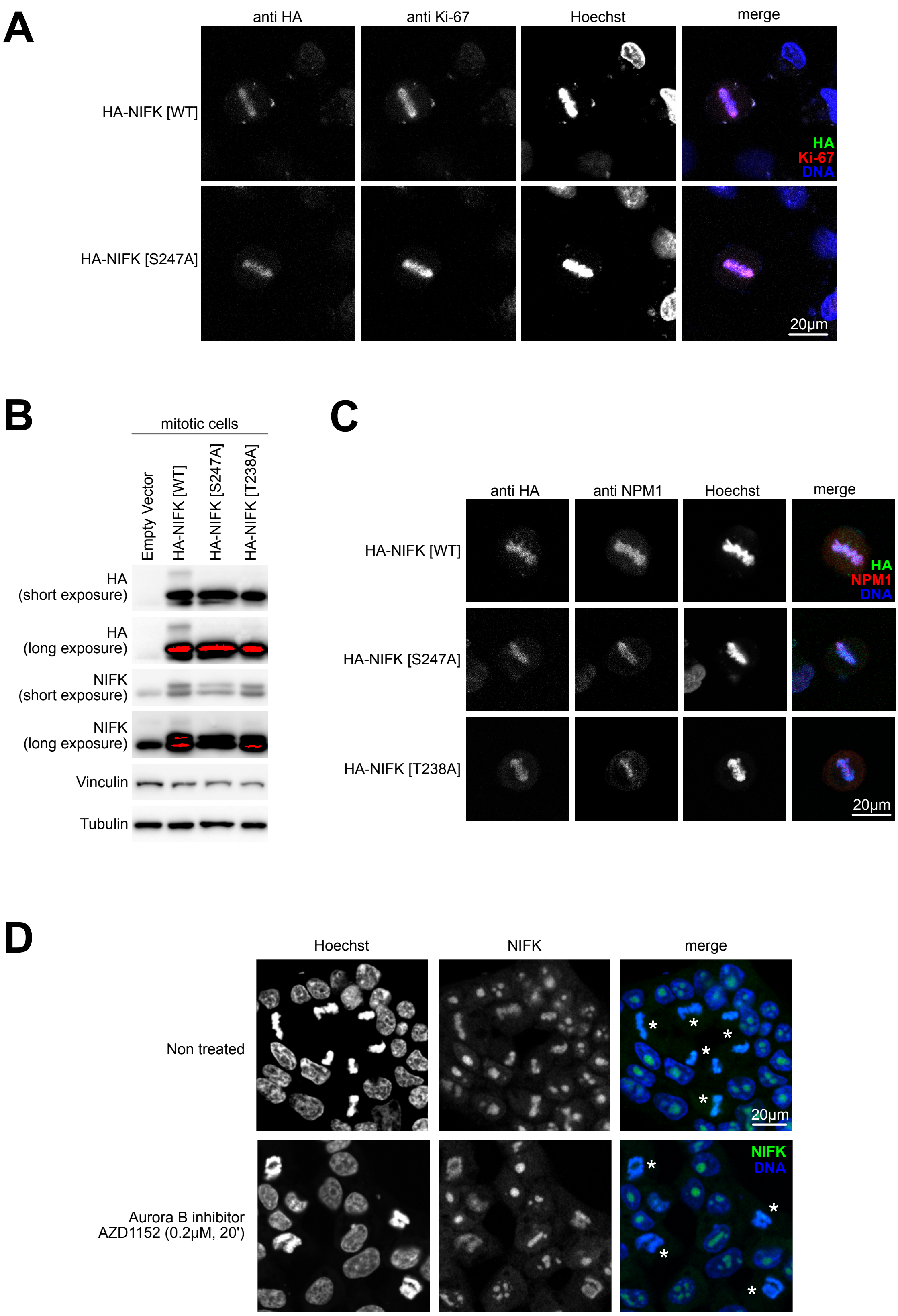
A
anti HA
anti Ki-67
Hoechst
merge
HA-NIFK [WT]
HA-NIFK [S247A]
HA
Ki-67
DNA
20µm
B
mitotic cells
Empty Vector
HA-NIFK [WT]
HA-NIFK [S247A]
HA-NIFK [T238A]
HA (short exposure)
HA (long exposure)
NIFK (short exposure)
NIFK (long exposure)
Vinculin
Tubulin
C
anti HA
anti NPM1
Hoechst
merge
HA-NIFK [WT]
HA-NIFK [S247A]
HA-NIFK [T238A]
HA
NPM1
DNA
20µm
D
Hoechst
NIFK
merge
Non treated
Aurora B inhibitor AZD1152 (0.2µM, 20')
NIFK
DNA
20µm

Fig. S3. NIFK phosphorylation at Ser234, Ser238 or Ser247 are not required for association with mitotic chromosomes

(A) Both HA-NIFK[WT] and HA-NIFK[S247A] localize to mitotic chromosomes in HeLa cells. Asynchronously growing HeLa cells transfected with the corresponding expression constructs were stained with anti-HA antibody (green), anti-Ki67 antibody (red) and with Hoechst 33342 (blue) to stain DNA. All mitotic cells that were observed (n>50) have this pattern.

(B) Mutation of either S247 or S238 to alanine abolishes most of the mitotic phosphorylations on NIFK. HeLa cells were transfected with the indicated constructs and then synchronized in mitosis via an overnight thymidine block, followed by release into medium containing Cdk1 inhibitor for 8.5 hours, followed by release into mitosis for 25 minutes.

(C) HA-NIFK[WT], HA-NIFK[S247A], and HA-NIFK[S238A] all localize to mitotic chromosomes in HeLa cells. Asynchronously growing HeLa cells transfected with the corresponding expression constructs were stained with anti-HA antibody (green), anti-NPM antibody (red) and with Hoechst 33342 (blue) to stain DNA. All mitotic cells that were observed (n>50) have this pattern.

(D) Aurora B inhibition does not displace NIFK from the perichromosomal layer. Asynchronous cells were treated with Aurora B inhibitor AZD1152 for 20 minutes and then immunostained for NIFK. Mitotic cells (asterisks) were identified morphologically due to condensed chromatin. Aurora B inhibition produces an expected, characteristic chromosomal phenotype caused by premature entry into anaphase. NIFK is localized to mitotic chromosomes also upon Aurora B inhibition, which significantly blunts NIFK phosphorylation.

Journal of Cell Science • Supplementary information

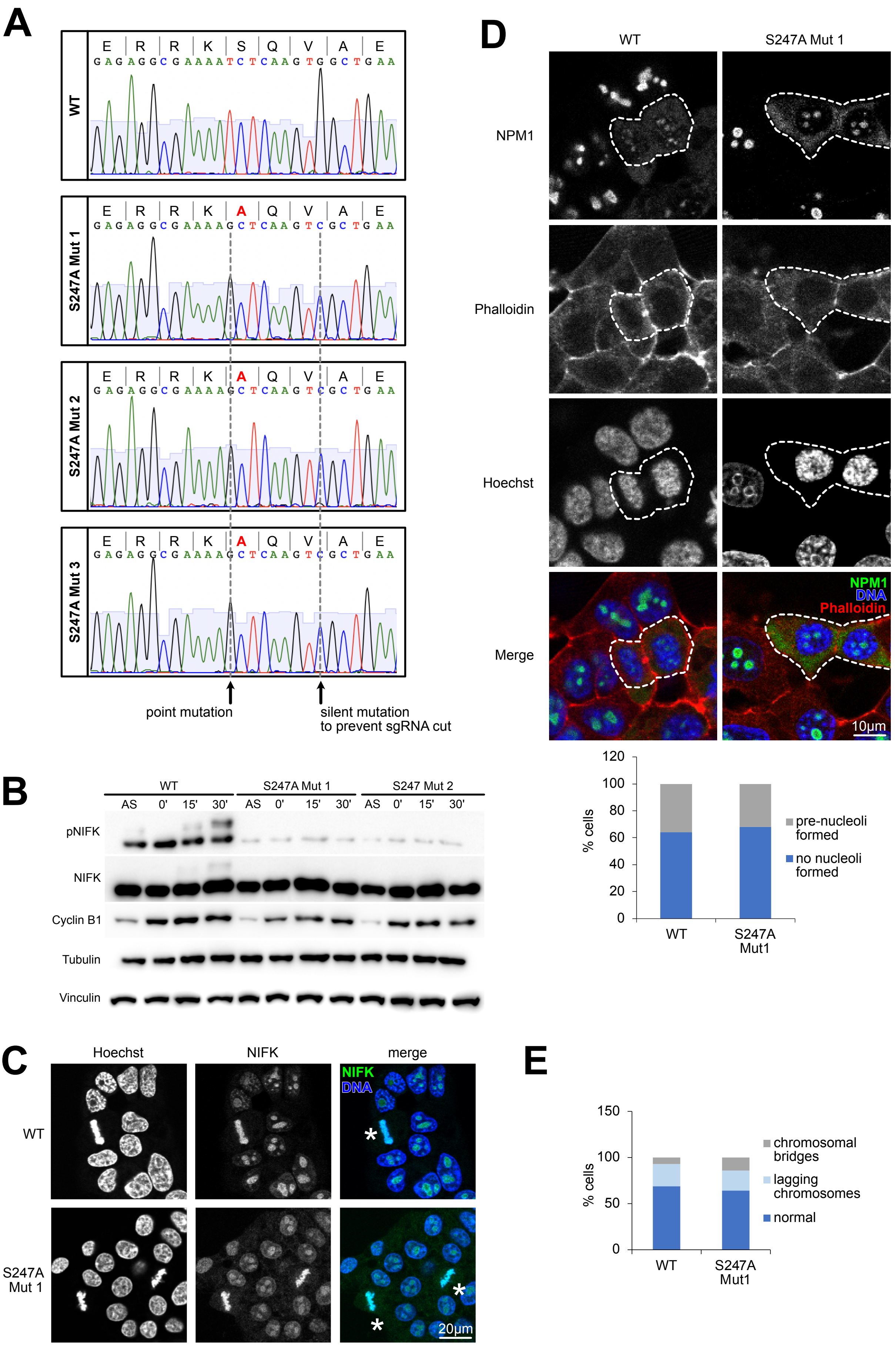

Journal of Cell Science • Supplementary information

Fig. S4. Characterization of HEK293T cells with a homozygous S247A mutation in the endogenous NIFK locus

(A) Sequencing traces of genomic DNA surrounding the codon coding for NIFK S247 from wildtype cells and cells that were genome edited using CRISPR/Cas9 and homologous directed recombination. Note that the genome editing was designed to introduce a serine-to-alanine mutation at position 247 as well as a silent mutation in Val249 which prevents the sgRNA from re-cutting after the genome editing event. Three independent lines with homozygous mutations were identified.

(B) NIFK[S247A] does not shift up during mitosis. Control and S247A mutant HEK293 cells were synchronized via an overnight thymidine block, followed by release into medium containing a CDK1 inhibitor to arrest cells at the entry into mitosis, followed by release into mitosis by washout of the CDK1 inhibitor. Samples were collected at the indicated timepoints after release into mitosis. "AS" = asynchronous control cells. Representative of 3 biological replicates.

(C) NIFK localizes to mitotic chromosomes in both control cells and cells carrying the S247A mutation. Asynchronous cells were fixed and immunostained for NIFK. Mitotic cells are identified morphologically by the condensed chromatin.

(D) Cells homozygous for NIFK[S247A] do not display a defect in nucleolar reformation after mitosis. Asynchronous control or NIFK[S247A] mutant cells were stained for NPM1 to mark nucleoli, with phalloidin to mark F-actin and with Hoechst to mark DNA. Telophase cells were identified by the presence of condensed chromatin in two neighboring cells that have reformed nuclei but

Journal of Cell Science • Supplementary information

are still connected by an actin-rich midbody. Bottom panel: 100 telophase cells were scored for the presence of NPM1 speckles ("pre-nucleoli").

(E) Cells homozygous for NIFK[S247A] display mildly increased numbers of late anaphase/telophase cells with chromosomal bridges. Asynchronous cells were fixed with formaldehyde, stained with Hoechst for DNA. Late anaphase/telophase cells were identified by chromosomal morphology and scored for the presence of lagging strands or chromosomal bridges.

Journal of Cell Science • Supplementary information

Table S1. Nucleic acid sequences

<u>Oligos</u>	
NIFK -topo fwd	ttagcgtcggcggctttt
NIFK topo_rev	agtgaaatacaaagtccactctca
NIFK EcoRI fwd	ccgggaattcaccatggcgactttttct
NIFK NotI rev	ccggGCGGCCGCtcactgattgctgct
NIFK S247A fwd	TTGGAGAGGCGAAAAGCCCAAGTGGCTGAACTG
NIFK S247A rev	CAGTTCAGCCACTTGGGCTTTTCGCCTCTCCAA
NIFK S238A fwd	CCCACACCAGTTTGTgccCCAACATTTTTGGAG
NIFK S238A rev	CTCCAAAAATGTTGGgg**c**ACAAACTGGTGTGGG
NIFK aa226fwd	GGccatggagaagactgtggata
NIFK S247A genotyping	GGGGCACAACTTGTAAAAATAGAGG
	TGCTGCTTCTTCGTCTTTTTTTCCG
<u>siRNAs</u>	
NIFK siRNA 1	GAAACGGUAUAAUCGGAAU
NIFK siRNA 2	CCACCUACCUAACCUACUU
<u>Genome editing</u>	
HDR repair template	/Alt-R-HDR1/C*A* TGT TTT ACA TTT ATG TAT TAT AAT CTT TAC TTG TTG AAT CCA GGG CCC CAC ACC AGT TTG TAC ACC AAC ATT TTT GGA GAG GCG AAA AGC TCA AGT CGC TGA ACT GAA TGA TGA TGA TAA AGA TGA TGA AAT AGT TTT CAA ACA GCC CAT ATC CTG TGT AAA AGA AGA AAT ACA AGA GAC* T*C/Alt- R-HDR2/
sgRNA	mG*mG*mA* rGrArG rGrCrG rArArA rArUrC rUrCrA rArGrG rUrUrU rUrArG rArGrC rUrArG rArArA rUrArG rCrArA rGrUrU rArArArArUrArArGrGrCrUrArGrUrCrCrGrU rUrArUrCrArArCrUrU rGrArA rArArA rGrUrG rGrCrArCrCrG rArGrU rCrGrG rUrGrC mU*mU*mU* rU
sgRNA target site	ATAGAGAGGAAAATCTCAAG
