# Supplementary material for: Stress- and mitosis-dependent phosphorylation of NIFK on Ser247
Source: J Cell Sci. 2026 Aug 27;139(16):jcs264641. doi: 10.1242/jcs.264641 (PMC13571814; doi:10.1242/jcs.264641)
Supplement: Supplementary file 2 [file joces-139-264641_review_history.pdf]

Stress- and mitosis-dependent phosphorylation of NIFK on Ser247

Sibylle Schleich and Aurelio A. Teleman
DOI: 10.1242/jcs.264641

Editor: Renata Basto

Review timeline
Original submission: 3 December 2025
Editorial decision: 5 January 2026
First revision received: 16 June 2026
Accepted: 7 July 2026

Original submission

First decision letter

MS ID#: jcs.264641

MS TITLE: Stress and mitosis-dependent phosphorylation of NIFK on Ser247 serves as a priming site for other phosphorylations

AUTHORS: Aurelio Teleman; Sibylle Schleich
ARTICLE TYPE: Short Report

Dear Dr Teleman,

We have now reached a decision on the above manuscript.

As you will see, the reviewers raise a number of substantial criticisms that prevent me from accepting the paper at this stage. They suggest, however, that a revised version might prove acceptable, if you can address their concerns. If you think that you can deal satisfactorily with the criticisms on revision, I would be pleased to see a revised manuscript. We would then return it to the reviewers. I agree with the points raised by the reviewers. It will be essential to show western blot quantifications in addition to statistical analysis along with the information concerning the number of replicates. Concerning the microscopy data, please do not forget to add scale bars and to include the fluorescence intensity quantifications.

Reviewer 1

SUMMARY OF THE ADVANCE MADE IN THIS PAPER AND ITS POTENTIAL SIGNIFICANCE TO THE FIELD

Overall, I believe the authors drew appropriate conclusions based on the data shown and not over extrapolating it. I also enjoyed seeing the schematics drawn by authors while reading the manuscripts, so I can easily follow the thinking process of the authors.

SUGGESTIONS TO AUTHORS

Major comments [Please request additional experiments only if they are essential for supporting the conclusions; authors should be encouraged to highlight any claims that are preliminary or speculative, or to discuss any pitfalls or alternative interpretations in a 'Limitations' section]

© 2026. Published by The Company of Biologists under the terms of the Creative Commons Attribution License (https://creativecommons.org/licenses/by/4.0/).

One major criticism I have and I believe is the biggest weakness of the manuscript is the lack of quantifications for all the western blots and phosphorylation studies. Without quantifications, I worry this manuscript would not be publishable at this current format. It is also unclear how many times each experiments were repeated to show consistent results. However, I do believe quantifications should be easily produced by the authors. Be able to show statistical significance would really solidify the conclusion

Minor comments

None

Reviewer 2

SUMMARY OF THE ADVANCE MADE IN THIS PAPER AND ITS POTENTIAL SIGNIFICANCE TO THE FIELD

Teleman and Schleich show phosphorylation of NIFK1 S247 in response to stress and during mitosis. They identify MSK1 and Aurora B as likely kinases responsible for these phosphorylation events, respectively. Furthermore, they show that the previously described hyperphosphoryation during mitosis depends on S247 phosphorylation. They address whether S247 phosphorylation affects the localization of NIFK to the perichromosomal layer and argue that it does not.

The paper presents intriguing data but overall it appears rather fragmented. The authors attempt to investigate both the stress- induced and the mitotic phosphorylation events, but neither is explored in sufficient depth, and the functional significance of these events remains unclear. I appreciate that these are distinct research areas and a comprehensive analysis of all possible consequences would be outside the scope, but the paper would be much improved by pursuing one or the other observation and exploring it more thoroughly (see major comments for examples). In addition, I find the text somewhat confusing; while many individual parts are well written, the paper lacks a cohesive narrative.

SUGGESTIONS TO AUTHORS

Major comments

1) The authors introduce Ki-67 and NIFK1 in the context of the perichromosomal layer and nucleolar function, and then report that NIFK1 S247 was identified in a proteomics screen aimed to identify novel GCN2 substrates. However, the role of GCN2 in ribosome biogenesis is not introduced and relevant papers (eg Piecyk et al 2024) are not cited.

The end of the Introduction "Given NIFK's established role in ribosome biogenesis, we hypothesized that NIFK may represent a previously unrecognized substrate through which GCN2 modulates ribosome-associated functions" rather suggested an investigation of ribosome biogenesis and nucleolar function to follow. This is one example when the narrative is confusing, because instead, the first Results chapter shows that NIFK is phosphorylated on Ser247 by MSK1 in response to ribotoxic stress.

A role of NIFK1 S247 phosphorylation in ribosome biogenesis is discussed and discarded based on published data that while NIFK-deficient cells have a defect in cell proliferation, this is rescued by NIFK transgene lacking the Ki-67-interaction domain including S247 (Pan et al 2015). While these observations indeed argue against an essential role in nucleolar function, Pan et al report only a partial rescue by the ΔK mutant. Thus, a role for S247 phosphorylation for example in the reorganization of the nucleoli during mitosis (disassembly and reformation) cannot be excluded and could be easily investigated. While I appreciate that the nucleolar organization might not be the authors' major focus, but this would be one possible avenue of investigation.

2) The authors show NIFK1 S247 phosphorylation in mitosis by Aurora B. These conclusions are well supported by the data. Addressing the significance of the mitotic phosphorylation would enhance the paper and could be another possibility for a deeper analysis. Are there mitotic errors, lagging chromosomes, micronuclei, increased cell death….. in cells expressing only the NIFK1 S247A or S247D mutant? Does NIFK localize to the perichromosomal layer when Aurora B is inhibited?

© 2026. Published by The Company of Biologists under the terms of the Creative Commons Attribution License (https://creativecommons.org/licenses/by/4.0/).

Furthermore, since the phosphorylation of NIFK was found to be GCN2-dependent in the proteomics screen, the role of GCN2 in mitosis (Stonyte et al 2023) should have been considered, even if the screen was designed based on amino-acid starvation.

3) Another possible focus could be the stress-induced phosphorylation of NIFK1 S247. The authors use anisomycin and conclude that the stress triggering phosohorylation is ribotoxic stress. While anisomycin can cause ribotoxic stress, additional stress-inducing agents should be used to confirm this model. The conseqences on survival, nucleolar structure, ribosome biogenesis… could be investigated using the S247A mutant.

4) A general comment regarding the use of drugs: the concentrations of all the drugs used in the paper are rather high (some examples are provided under "minor comments"). While the experiments using these drugs seem to support the conclusions, such high concentrations raise concerns and should always be interpreted with caution.

Minor comments

1) The conditions of the proteomics screen are not described in detail. Assuming they were the same as the conditions used in the verification experiments (Fig S1), 2 hours starvation seems a bit on the short side and becomes very sensitive to cell numbers and washing conditions. Note that GCN2 appears to be phosphorylated in + AA in the presence of 3 μM A92, not in -AA; is something mislabelled, or is this a loading issue? eIF2α-P is induced by the 2-hour withdrawal of amino acids, not in GCN2i, as expected. However, loading control suggests that some of this increase is due to loading. Immunoblot showing total eIF2α is missing.

2) Overnight (16h) treatment with 100 ng/ml nocodazole is extremely harsh and has a severe impact on global translation. While the authors also use thymidine + CDKi block to address mitotic phosphorylation, nocodazole treatment as performed cannot simply be interpreted as mitotic synchronization, especially in light of the stress-induced phosphorylation of the same residue.

3) Fig1 B: NIFK phosphorylation is inhibited by SB202190 but p38 phosphorylation is apparently not, based on the immunoblot shown. The concentration of the inhibitor is shown as 10 μM, usual concentration is 1-5 μM.

4) "treatment of cells with anisomycin at a concentration that partially inhibits translation, and thereby promotes ribosome collisions" translation is not measured, no reference is provided.

5) In Fig 2 they show NIFK1 phosphorylation by immunoblotting in synchronized cells. The extent of the synchrony and timing through mitosis should be shown (eg count mitotic cells, western or flowcytometry for H3-S10-P…). Confirming this finding in asynchronous cells by immunofluorescence is a very good initiative, but quantification should be shown in addition to representative images.

6) Having said that, the verification of the p-NIFK(S247) antibody in immunofluorescence is not entirely complete. While it appears that it does recognize the target epitope, a negative control, such as cells only expressing the S247A mutant, is necessary to exclude non-specific signals. In mitosis particularly many proteins are phosphorylated and a number of phospho-specific antibodies show non-specific signals.

7) Fig S2 they use AZD1152 at 2 μM BUT in HeLa cells, 100 nM to 500 nM is most commonly used for mitotic arrest or studying the impact of Aurora B inhibition.

8) Page 12 "spatial and temporal pattern of Aurora B activity in a mitotic cell (Fuller et al., 2008) matches the pattern that we observe for p-NIFK(S247) during mitosis (Fig. 2B" is not quite correct. Fuller et al show spatial Aurora B gradient in anaphase, while Fig 2B shows p-NIFK(S247)-positive cells from prometaphase to anaphase

9) The authors claim that S247 phosphorylation is not required for the localization of NIFK to the perichromosomal layer. However, they also show that overexpressed HA-NIFK co-immunoprecipitates endogenous NIFK (Supplementary Fig. 2D) suggesting dimerization, which raises

© 2026. Published by The Company of Biologists under the terms of the Creative Commons Attribution License (https://creativecommons.org/licenses/by/4.0/).

the question whether the localization they observe might be driven by the endogenous wild-type component of the dimers. These experiments should be repeated using cells that only express the mutant protein (eg knocking down the endogenous protein in cells expressing siRNA resistant HA-tagged wt or mutant protein)

First revision

Author response to reviewers' comments

Reviewer 1

Overall, I believe the authors drew appropriate conclusions based on the data shown and not over extrapolating it. I also enjoyed seeing the schematics drawn by authors while reading the manuscripts, so I can easily follow the thinking process of the authors.

SUGGESTIONS TO AUTHORS

Major comments [Please request additional experiments only if they are essential for supporting the conclusions; authors should be encouraged to highlight any claims that are preliminary or speculative, or to discuss any pitfalls or alternative interpretations in a 'Limitations' section]

One major criticism I have and I believe is the biggest weakness of the manuscript is the lack of quantifications for all the western blots and phosphorylation studies. Without quantifications, I worry this manuscript would not be publishable at this current format. It is also unclear how many times each experiments were repeated to show consistent results. However, I do believe quantifications should be easily produced by the authors. Be able to show statistical significance would really solidify the conclusion

We thank the reviewer for the positive comments and constructive suggestions. We have now added quantifications to the main figures, which provide all the main cornerstone conclusions of the manuscript. All the original conclusions were statistically significant. For the supplementary figures, in some cases we only have 2 biological replicates, so instead we added information about how many biological replicates were performed to the figure legends. We have also added a large amount of new data in response to the comments by Reviewer 2. We hope the reviewer finds the manuscript improved.

Minor comments

None

Reviewer 2

Teleman and Schleich show phosphorylation of NIFK1 S247 in response to stress and during mitosis. They identify MSK1 and Aurora B as likely kinases responsible for these phosphorylation events, respectively. Furthermore, they show that the previously described hyperphosphoryation during mitosis depends on S247 phosphorylation. They address whether S247 phosphorylation affects the localization of NIFK to the perichromosomal layer and argue that it does not.

The paper presents intriguing data but overall it appears rather fragmented. The authors attempt to investigate both the stress- induced and the mitotic phosphorylation events, but neither is explored in sufficient depth, and the functional significance of these events remains unclear. I appreciate that these are distinct research areas and a comprehensive analysis of all possible consequences would be outside the scope, but the paper would be much improved by pursuing one or the other observation and exploring it more thoroughly (see major comments for examples). In addition, I find the text somewhat confusing; while many individual parts are well written, the paper lacks a cohesive narrative.

© 2026. Published by The Company of Biologists under the terms of the Creative Commons Attribution License (https://creativecommons.org/licenses/by/4.0/).

SUGGESTIONS TO AUTHORS

Major comments

1) The authors introduce Ki-67 and NIFK1 in the context of the perichromosomal layer and nucleolar function, and then report that NIFK1 S247 was identified in a proteomics screen aimed to identify novel GCN2 substrates. However, the role of GCN2 in ribosome biogenesis is not introduced and relevant papers (eg Piecyk et al 2024) are not cited.

The end of the Introduction "Given NIFK's established role in ribosome biogenesis, we hypothesized that NIFK may represent a previously unrecognized substrate through which GCN2 modulates ribosome-associated functions" rather suggested an investigation of ribosome biogenesis and nucleolar function to follow. This is one example when the narrative is confusing, because instead, the first Results chapter shows that NIFK is phosphorylated on Ser247 by MSK1 in response to ribotoxic stress.

We agree - we had originally presented our data in a historical context to explain why we started studying S247 phosphorylation, however this caused confusion. We have now removed GCN2 and all related data entirely from the manuscript because our data do not support a role for GCN2 in NIFK S247 phosphorylation. We hope this makes the narrative more linear.

A role of NIFK1 S247 phosphorylation in ribosome biogenesis is discussed and discarded based on published data that while NIFK-deficient cells have a defect in cell proliferation, this is rescued by NIFK transgene lacking the Ki-67-interaction domain including S247 (Pan et al 2015). While these observations indeed argue against an essential role in nucleolar function, Pan et al report only a partial rescue by the ΔK mutant. Thus, a role for S247 phosphorylation for example in the reorganization of the nucleoli during mitosis (disassembly and reformation) cannot be excluded and could be easily investigated. While I appreciate that the nucleolar organization might not be the authors' major focus, but this would be one possible avenue of investigation.

We thank the reviewer for this comment. We have now done two things:

1. In agreement with what the reviewer writes above, we have reworded the text. In the Introduction, we now write:

> "Functional studies demonstrated that cells expressing a NIFK mutant lacking the Ki-67 binding domain proliferate at roughly half the speed of control cells (Pan et al., 2015). Thus, the interaction of NIFK with Ki-67 does not appear to be essential for cell viability, but it may affect the function of NIFK in ribosome biogenesis and cell cycle progression. "

The results section now says:

> "Thus the Ki-67 interaction domain does not appear to be <u>absolutely required</u> for NIFK to promote ribosome biogenesis."

2. As suggested, we have studied the effect of S247 phosphorylation on nucleolar organization during mitosis. This was not as easy as one might think. To study this, one needs a system where the endogenous NIFK is replaced with a S247A mutant. One way to achieve this is to generate a NIFK knockout line and then reconstitute it with either wildtype or mutant NIFK. We tried generating NIFK knockout cells several times in the past but were unsuccessful, suggesting that NIFK is required for cell viability. A second approach to bypass the viability problem is to introduce an inducible degron into the endogenous NIFK locus, to provide wildtype or mutant NIFK via a stable transgene, and then to induce degradation of the endogenous NIFK. We tried this, but did not obtain a line with a homozygous degron insertion despite screening many clones. As a third approach, we tried knocking down endogenous NIFK with siRNAs targeting the 3'UTR and then re-expressed NIFK from a transgene lacking a 3'UTR, but we were not able to reconstitute cell viability with this approach. Finally, we tried a genome editing approach. We used CRISPR/Cas9 to introduced a Ser274Ala mutation into the endogenous NIFK locus. This worked, and we obtained three independent lines with homozygous insertions (Suppl. Fig. 4A). We then analyzed nucleolar disassembly and reformation during mitosis, but do not see any phenotypes. By prometaphase, both

© 2026. Published by The Company of Biologists under the terms of the Creative Commons Attribution License (https://creativecommons.org/licenses/by/4.0/).

control and S247A mutant cells lose their nucleoli and NPM1 can be detected on the perichromosomal layer (Suppl. Fig. 4B). At the end of mitosis, the dynamics of nucleolar reformation is the same in S247A mutant and control cells - we can start seeing initial NPM1 speckles in roughly 40% of telophase cells (Suppl. Fig. 4D). Also in interphase cells nucleolar morphology appears to be the same in S247A mutants and controls (Suppl. Fig. 4B). Hence we do not see any phenotypes in terms of nucleolar morphology.

Please note, however, that this does not exclude a possible role for this phosphorylation in regulating ribosome biogenesis. We note this explicitly in the Discussion, as well as the fact that we are happy to distribute the S247A line to anyone who would like to study it further, and perhaps has more expertise than we do in this topic.

2) The authors show NIFK1 S247 phosphorylation in mitosis by Aurora B. These conclusions are well supported by the data. Addressing the significance of the mitotic phosphorylation would enhance the paper and could be another possibility for a deeper analysis. Are there mitotic errors, lagging chromosomes, micronuclei, increased cell death….. in cells expressing only the NIFK1 S247A or S247D mutant? Does NIFK localize to the perichromosomal layer when Aurora B is inhibited? Furthermore, since the phosphorylation of NIFK was found to be GCN2-dependent in the proteomics screen, the role of GCN2 in mitosis (Stonyte et al 2023) should have been considered, even if the screen was designed based on amino-acid starvation.

We now provide new data showing that:

1. NIFK still localizes to the perichromosomal layer when Aurora B is inhibited (Suppl. Fig. 3D).
2. Consistent with that, using the S247A genome-editing line, we find that NIFK[S247A] mutant also localizes to the perichromosomal layer (Suppl. Fig. 4B).
3. Using the genome-edited line, we do see some more chromosomal bridges in the S247A cells compared to control cells (Suppl. Fig. 4C) however we do not see more micronuclei nor a proliferation defect in the S247 line, so we are unsure whether this is a significant phenotype.
4. We do find a delay in mitotic entry in NIFK[S247A] cells. If we synchronize cells by performing a thymidine block, then releasing the cells into medium containing a Cdk1 inhibitor to synchronize them at mitotic entry, and then release the cells into mitosis by washing out the Cdk1 inhibitor, we find that all three S247A lines are delayed (Fig. 5). Thus it looks like phosphorylation on S247 does have a functional role in mitosis.

Regarding GCN2, we do not believe NIFK S247 is phosphorylated by GCN2 and therefore removed all GCN2-related data from the manuscript.

3) Another possible focus could be the stress-induced phosphorylation of NIFK1 S247. The authors use anisomycin and conclude that the stress triggering phosohorylation is ribotoxic stress. While anisomycin can cause ribotoxic stress, additional stress-inducing agents should be used to confirm this model. The conseqences on survival, nucleolar structure, ribosome biogenesis… could be investigated using the S247A mutant.

We agree with the reviewer that any stress that activates p38 should cause S247 phosphorylation, and we originally worded our manuscript too narrowly. We have now tested additional stresses and provide new data showing that also UV irradiation and TNF□, both of which activate p38, lead to NIFK S247 phosphorylation in an MSK1-dependent manner (new Figure 2A-B). We have also reworded the manuscript to indicate that S247 is phosphorylated more broadly in response to stress.

Regarding nucleolar structure, we added new data showing no phenotypes in the S247A line - please see our reply above to point #1.

Regarding ribosome biogenesis, we were unable within the timeframe of this revision to set up an assay for 28S and 5.8S rRNA maturation. In principle the S247A line would be a perfect tool to test this. We're happy to share the line with labs that specialize in ribosome biogenesis.

© 2026. Published by The Company of Biologists under the terms of the Creative Commons Attribution License (https://creativecommons.org/licenses/by/4.0/).

4) A general comment regarding the use of drugs: the concentrations of all the drugs used in the paper are rather high (some examples are provided under "minor comments"). While the experiments using these drugs seem to support the conclusions, such high concentrations raise concerns and should always be interpreted with caution.

Please see our responses below to the minor comments.

Minor comments

1) The conditions of the proteomics screen are not described in detail. Assuming they were the same as the conditions used in the verification experiments (Fig S1), 2 hours starvation seems a bit on the short side and becomes very sensitive to cell numbers and washing conditions. Note that GCN2 appears to be phosphorylated in + AA in the presence of 3 μM A92, not in -AA; is something mislabelled, or is this a loading issue? eIF2α-P is induced by the 2-hour withdrawal of amino acids, not in GCN2i, as expected. However, loading control suggests that some of this increase is due to loading. Immunoblot showing total eIF2α is missing.

In the past we did extensive time courses with amino acid removal on GCN2 activation and one can already see robust GCN2 activation at 1 hour, so 2 hours should be safe (e.g. Fig 2A in Figlia et al. mTORC1 senses glutamine and other amino acids through GCN2. EMBO J 2025, PMID 40691417). We also do not think the samples were swapped in the figure referenced by the reviewer because the -AA samples had low p-S6K and high p-eIF2□ which was blocked by the A-92 inhibitor, as one would expect. Nonetheless, as discussed above we have removed all GCN2 data to focus the story on the positive results and to make the narrative more linear.

2) Overnight (16h) treatment with 100 ng/ml nocodazole is extremely harsh and has a severe impact on global translation. While the authors also use thymidine + CDKi block to address mitotic phosphorylation, nocodazole treatment as performed cannot simply be interpreted as mitotic synchronization, especially in light of the stress-induced phosphorylation of the same residue.

We agree fully with the reviewer that the two treatments are not equivalent - the nocodazole treatment arrests cells in mitosis for hours, activates the spindle-assembly checkpoint, and causes global inhibition of translation, whereas the thymidine + CDK1 block/release protocol yields cells that are progressing through mitosis with minimal stress. For this reason, in the manuscript we refer to the nocodazole-treated cells as "arrested in mitosis" and the CDK1 block/release cells as "progressing through mitosis", and we've clearly marked in the figures which method was used where.

Importantly, in Fig 3A, we show that S247 is phosphorylated in non-stressed cells progressing through mitosis (i.e. thymidine block - CDK block - release). Consistent with this, in Suppl. Fig. 2A we show that MSK inhibition does not block phosphorylation of S247 in mitotic cells that are not stressed, indicating that phosphorylation of S247 during mitosis is not due to stress and there are two separate branches that can cause S247 phosphorylation.

That said, the panels where we used nocodazole could potentially reflect a combination of activity of the two branches - mitosis and stress. However, in Fig. 3B, we find that S247 phosphorylation in nocodazole-treated cells is abolished with an AurB inhibitor, suggesting that our nocodazole treatment is not causing p38 activation in this setup. (Note that although we use nocodazole in Fig 3B, we use non-stress conditions in Suppl. Fig. 2B, which shows the same result).

3) Fig1 B: NIFK phosphorylation is inhibited by SB202190 but p38 phosphorylation is apparently not, based on the immunoblot shown. The concentration of the inhibitor is shown as 10 μM, usual concentration is 1-5 μM.

SB202190 inhibits p38 by occupying the ATP pocket, but it does not inhibit the upstream kinases that phosphorylate p38 on T180/182. As a result, SB202190 causes inhibition of p38 activity without

© 2026. Published by The Company of Biologists under the terms of the Creative Commons Attribution License (https://creativecommons.org/licenses/by/4.0/).

necessarily blocking phosphorylation on T180/182 (PMID 10512765). (Apparently SB202190 can also affect T180/182 phosphorylation in some cases but this is context dependent.)

We selected 10μM for SB202190 based on the range we found in the literature, which is 1-10μM with 10μM being used quite frequently, e.g. https://pmc.ncbi.nlm.nih.gov/articles/PMC7264467/

4) "treatment of cells with anisomycin at a concentration that partially inhibits translation, and thereby promotes ribosome collisions" translation is not measured, no reference is provided.

Thanks for catching that - we have now added a reference to the paper from Rachel Green where we got this from (PMID 32610081).

5) In Fig 2 they show NIFK1 phosphorylation by immunoblotting in synchronized cells. The extent of the synchrony and timing through mitosis should be shown (eg count mitotic cells, western or flowcytometry for H3-S10-P…). Confirming this finding in asynchronous cells by immunofluorescence is a very good initiative, but quantification should be shown in addition to representative images.

(Please note Fig. 2 is now Fig. 3 in the revised version). We have now added Fig. 3B which shows the percentage of pH3+ cells at the various timepoints used in Fig. 3A.

Regarding the immunofluorescence images with the p-NIFK antibody, please see below the reply to the next question:

6) Having said that, the verification of the p-NIFK(S247) antibody in immunofluorescence is not entirely complete. While it appears that it does recognize the target epitope, a negative control, such as cells only expressing the S247A mutant, is necessary to exclude non-specific signals. In mitosis particularly many proteins are phosphorylated and a number of phospho-specific antibodies show non-specific signals.

We agree with the reviewer - although we extensively validated the p-NIFK(S247) antibody signal for western blots, we did not validate it for cell immunostaining, because we previously lacked the necessary tools. Now that we were able to genome edit the endogenous NIFK locus and generate cells expressing only NIFK[S247A] we were able to do this important control experiment. Surprisingly, however, we did not observe a reduction in signal in the S247A cells. Thus the immunostaining signal is mainly detecting NIFK regardless of phosphorylation state. We have therefore removed that panel from the manuscript, and now the manuscript relies only on western blots, for which the specificity of the phospho-antibody is validated.

7) Fig S2 they use AZD1152 at 2 μM BUT in HeLa cells, 100 nM to 500 nM is most commonly used for mitotic arrest or studying the impact of Aurora B inhibition.

Thanks for catching that. We mislabeled the figures and we did indeed use 0.2μM. To make absolutely sure, we have repeated these experiments during the revisions with 0.2μM and the results reproduced. We have now corrected the labeling in the figure.

8) Page 12 "spatial and temporal pattern of Aurora B activity in a mitotic cell (Fuller et al., 2008) matches the pattern that we observe for p-NIFK(S247) during mitosis (Fig. 2B" is not quite correct. Fuller et al show spatial Aurora B gradient in anaphase, while Fig 2B shows p-NIFK(S247)-positive cells from prometaphase to anaphase

Since we removed the immunostaining data, we have also removed this sentence.

© 2026. Published by The Company of Biologists under the terms of the Creative Commons Attribution License (https://creativecommons.org/licenses/by/4.0/).

9) The authors claim that S247 phosphorylation is not required for the localization of NIFK to the perichromosomal layer. However, they also show that overexpressed HA-NIFK co-immunoprecipitates endogenous NIFK (Supplementary Fig. 2D) suggesting dimerization, which raises the question whether the localization they observe might be driven by the endogenous wild-type component of the dimers. These experiments should be repeated using cells that only express the mutant protein (eg knocking down the endogenous protein in cells expressing siRNA resistant HA-tagged wt or mutant protein)

This is a good point. Now that we were able to do the genome editing and obtained cells homozygously expressing only NIFK[S247A], we repeated this experiment and nonetheless NIFK[S247A] still localizes to the perichromosomal layer (Suppl. Fig. 4B).

Second decision letter

MS ID#: jcs.264641R1

MS Title: Stress and mitosis-dependent phosphorylation of NIFK on Ser247

Authors: Aurelio Teleman; Sibylle Schleich
Article Type: Research Article

Dear Dr Teleman,

I am happy to tell you that your manuscript has been accepted for publication in Journal of Cell Science, pending standard publication integrity checks.

Reviewer 2

SUMMARY OF THE ADVANCE MADE IN THIS PAPER AND ITS POTENTIAL SIGNIFICANCE TO THE FIELD

The revised manuscript identifies Ser247 as a stress- and mitosis-responsive phosphorylation site on NIFK and provides convincing evidence that this phosphorylation primes mitotic hyperphosphorylation and contributes to timely mitotic entry.
The additional functional analyses provide important mechanistic insight, making this a valuable contribution to our understanding of NIFK regulation.

SUGGESTIONS TO AUTHORS

I thank the authors for a thorough and thoughtful revision. The revised manuscript is substantially improved and addresses all my concerns.

I particularly commend the authors for their perseverance in pursuing the S247A cell line and carrying out the additional genome-editing-based experiments.
The cell line proved to be a valuable tool, allowing the authors to directly test several mechanistic hypotheses and clarify key aspects of NIFK Ser247 function. These new data result in a much sharper and more coherent story.

© 2026. Published by The Company of Biologists under the terms of the Creative Commons Attribution License (https://creativecommons.org/licenses/by/4.0/).
